# Supplementary material for: Trends in anxiety and depressive symptoms among adults in Norway based on eight population-based surveys from 1995 to 2024
Source: Discov Ment Health. 2026 Jul 2;6(1):168. doi: 10.1007/s44192-026-00522-4 (PMC13542007; doi:10.1007/s44192-026-00522-4)
Supplement: Supplementary file 1 [file 44192_2026_522_MOESM1_ESM.docx]

**Supplementary material**

Trends in symptoms of anxiety and depression among adults in Norway: Evidence from eight population-based surveys (1995-2024), European Journal of Epidemiology, Benedicte Kirkøen, Astrid MA Eriksen, Ann Ragnhild Broderstad, Anne Høye, Jonas Johansson, Børge Sivertsen, Elin Skretting Lunde, Steinar Krokstad, Eivind Aakhus, Ellen Melbye Langballe, Thomas Sevenius Nilsen, Marit Knapstad, Thomas Hansen, Martin Tesli, Anne Reneflot

Corresponding author

Benedicte Kirkøen, Department of Mental Health, Norwegian Institute of Public Health, Oslo, Norway

[benedicte.kirkoen@fhi.no](mailto:benedicte.kirkoen@fhi.no)

**Table S1.** Response rate for different age groups

| **Study** | **Year** | **Sample n*** | **Population** | **Response rate** |
| --- | --- | --- | --- | --- |
| **Living Conditions Survey.** Statistics Norway | 1998 | 6886 | National | **72%**  By age: not available |
|  | 2002 | 5313 | National | **64%**  By age:  16-24 years: 57%  25-44 year: 63%  45-66 years: 71%  67-79 year: 64% |
|  | 2005 | 4705 | National | **57%**  By age:  16-24 years:49%  25-44 year: 54%  45-66 years: 62%  67-79 year: 58% |
|  | 2008 | 4392 | National | **50%**  By age:  16-24 years: 37%  25-44 year: 45 %  45-66 years: 59%  67-79 year: 57% |
|  | 2012 | 3895 | National | **71%**  By age: not available |
|  |  |  |  |  |
|  | 2015 | 8104 | National | **59%**  By age:  16-24 years: 62%  25-44 year: 55%  45-66 years: 63%  67-79 year: 63%  80+ : 50% |
|  | 2019 | 7897 | National | **57%**  By age:  16-24 years: 55%  25-44 year: 54%  45-66 years: 61%  67-79 year: 62%  80+ : 48% |
| **Study** | **Year** | **Sample n*** | **Population** | **Response rate** |
| **The Trøndelag Health Study (HUNT)** | 1995-1997 | 62 444 | Trøndelag County, 20 years + | **70%**  By age(1)  (women-men)  19-29: 56-42%  30-39: 75-62%  40-49: 82-72%  50-59: 86-77%  60-69: 87-84%  70-79: 79-80%  80-89: 65-68% |
|  | 2006-2008 | 48 362 | Trøndelag County, 20 years + | **54%**  By age (2)  (women-men)  19-29: 38-26%  30-39: 51-36%  40-49: 64-49%  50-59: 71-61%  60-69: 74-66%  70-79: 67-66%  80-89: 39-45% |
|  | 2017-2019 | 52442 | Trøndelag County, 20 years + | **54%**  By age(3)  (women-men)  19-29: 43-31%  30-39: 53-38%  40-49: 59-45%  50-59: 66-54%  60-69: 70-64%  70-79: 68-65%  80-89: 54-55% |
| **Study** | **Year** | **Sample n*** | **Population** | **Response rate** |
| **Quality of life survey.**  Statistics Norway | 2020** | 17.529 | National | **44%**  By age:  18-24 years: 39,8%  25-44 year: 40,3%  45-66 years: 51,7%  67-79 year: 44,8%  80+ : 17,4% |
|  | 2021 | 17.544 | National | **44%**  By age:  18-24 years: 36,7%  25-44 year: 39,4%  45-66 years: 52,3%  67-79 year:48,1%  80+ : 19,1% |
|  | 2022 | 15.133 | National | **38%**  By age  18-24 years: 31%  25-44 year: 33,6%  45-66 years: 45,2%  67-79 year: 41,9%  80+ : 19% |
|  | 2023 | 17.972 | National | **45%**  By age  18-24 years: 40,1%  25-44 year: 43,1%  45-66 years: 52,3%  67-79 year:45,4%  80+ : 19,7% |
|  | 2024 | 17.229 | National | **43%**  By age  18-24 years: 38,1%  25-44 year: 40,7%  45-66 years: 50,7%  67-79 year:44,5%  80+ :21,2% |
| **Study** | **Year** | **Sample n*** | **Population** | **Response rate** |
| **Student’s Health and Wellbeing Study (SHoT)** | 2010 | 5 971 | Random sample of full-time students | **23%** |
|  | 2014 | 13523 | Random sample of full-time students | **29%** |
|  | 2018 | 49754 | Inviting all Norwegian full-time students | **31%** |
|  | 2021 | 59028 | Inviting all Norwegian full-time students | **34%** |
|  | 2022 | 52959 | Inviting all Norwegian full-time students | **35%** |
| **The Tromsø Study** | 2001 | 7021 | Regional. Tromsø municipality, 30+ | **78%**  By age:  (women-men)  30-34: 53-38%  35-39: 86-83%  40-49: 70-61%  50-59: 94-92%  60-69: 91-90%  70-79: 83-87%  80-87: 65-70% |
|  | 2007-2008 | 12 293 | Regional. Tromsø municipality, 30+ | **66%**  By age (4)  (women-men)  30-39: 54-38%  40-49: 65-56%  50-59: 75-66%  60-69: 80-74%  70-79: 66-69%  80-87: 37-36% |
|  | 2015-2016 | 20 422 | Regional. Tromsø municipality, 40+ | **65%**  By age(5)  (women-men)  40-49:65-55%  50-59: 72-65%  60-69: 75-71%  70-79: 68-69%  80-89: 40-51% |
| **Study** | **Year** | **Sample n*** | **Population** | **Response rate** |
| **SAMINOR** | 2003-2004 | 11304 | 24 municipalities in the North of Norway:  40-69 years | **64%**  By age  40-44:57%  45-49:61%  50-54:65%  55-59:66%  60-64:68%  65-69:66% |
|  | 2012 | 6776 | 24*** municipalities in the North of Norway:  40-69 years | **32%**  By age  40-49:28%  50-59: 33%  60-69: 35% |
| **FHUS Oslo** | 2020 | 8 865 | Oslo | **40%**  By age  Women -men  18-29: 32-20%  30-39: 41-29%  40-49: 50-39%  50-59: 56-46%  60-69: 63-52%  70-79: 61-59 %  80+: 27-43% |
|  | 2021 | 7 506 | Oslo | **35%**  By age  Women -men  18-29: 21-13%  30-39: 33-25%  40-49: 43-34%  50-59: 52-43%  60-69: 57-53%  70-79: 55-57 %  80+: 28-47% |
|  | 2024 | 43 848 | Oslo | **28%**  By age  Women- men  18-29: 24-15%  30-39: 29-21%  40-49: 31-24%  50-59: 39-31%  60-69: 44-36%  70-79: 50-47%  80+ 32-25% |
| **FHUS Agder** | 2019 | 28 000 | Agder | **46%**  By age  Women- men  18-29: 44-28%  30-39: 46-33%  40-49: 51-42%  50-59: 56-47%  60-69: 58-59%  70+: 47-56% |
|  | 2023 | 18 379 | Agder | **32%**  By age  Women- men  18-29: 25-12%  30-39: 33-20%  40-49: 37-26%  50-59: 44-32%  60-69: 46-41%  70-79: 43-48%  80+: 35-34% |

**References**

1.	Holmen J. The Nord-Trøndelag Health Study 1995-97 (HUNT 2). Norsk epidemiologi. 2003;13(1).

2.	Krokstad S, Langhammer A, Hveem K, Holmen T, Midthjell K, Stene T, et al. Cohort profile: the HUNT study, Norway. International journal of epidemiology. 2013;42(4):968-77.

3.	Åsvold BO, Langhammer A, Rehn TA, Kjelvik G, Grøntvedt TV, Sørgjerd EP, et al. Cohort profile update: the HUNT study, Norway. International journal of epidemiology. 2023;52(1):e80-e91.

4.	Eggen AE, Mathiesen EB, Wilsgaard T, Jacobsen BK, Njølstad I. The sixth survey of the Tromsø study (Tromsø 6) in 2007–08: collaborative research in the interface between clinical medicine and epidemiology: study objectives, design, data collection procedures, and attendance in a multipurpose population-based health survey. Scandinavian journal of public health. 2013;41(1):65-80.

5.	Hopstock LA, Grimsgaard S, Johansen H, Kanstad K, Wilsgaard T, Eggen AE. The seventh survey of the Tromsø Study (Tromsø7) 2015–2016: study design, data collection, attendance, and prevalence of risk factors and disease in a multipurpose population-based health survey. Scandinavian Journal of Public Health. 2022;50(7):919-29.
