# Supplementary material for: Trends in anxiety and depressive symptoms among adults in Norway based on eight population-based surveys from 1995 to 2024
Source: Discov Ment Health. 2026 Jul 2;6(1):168. doi: 10.1007/s44192-026-00522-4 (PMC13542007; doi:10.1007/s44192-026-00522-4)
Supplement: Supplementary file 2 [file 44192_2026_522_MOESM2_ESM.docx]

**Supplementary material**

Trends in symptoms of anxiety and depression among adults in Norway: Evidence from eight population-based surveys (1995-2024), European Journal of Epidemiology, Benedicte Kirkøen, Astrid MA Eriksen, Ann Ragnhild Broderstad, Anne Høye, Jonas Johansson, Børge Sivertsen, Elin Skretting Lunde, Steinar Krokstad, Eivind Aakhus, Ellen Melbye Langballe, Thomas Sevenius Nilsen, Marit Knapstad, Thomas Hansen, Martin Tesli, Anne Reneflot

Corresponding author

Benedicte Kirkøen, Department of Mental Health, Norwegian Institute of Public Health, Oslo, Norway

[benedicte.kirkoen@fhi.no](mailto:benedicte.kirkoen@fhi.no)

**Table 1a.** Mean score and percentage scoring above cut-off level for mental health problems by age group for women in the Living Conditions Survey (paper version).

| **Living Conditions Survey (paper version)** | | | | | | | | |
| --- | --- | --- | --- | --- | --- | --- | --- | --- |
| **Year** | **N=** | **m=** | **sd=** | **Reg. Coefficient comparing this year to the year prior** | **P=** | **Percentage scoring above ≥1.80** | **OR for being above cut of in this year compared to the year prior** | **P=** |
| **Women, 20-29 years** | | | | | | | | |
| 1998 | 613 | 1,37 | 0,55 |  |  | 17,94 |  |  |
| 2002 | 393 | 1,33 | 0,48 | -0.01 | 0.46 | 16,79 | 0.99 | 0.88 |
| 2005 | 370 | 1,39 | 0,57 | 0.03 | 0.11 | 18,92 | 1.07 | 0.47 |
| 2008 | 259 | 1,40 | 0,50 | 0.01 | 0.74 | 18,92 | 1.02 | 0.86 |
| 2012 | 238 | 1,55 | 0,64 | 0.06 | **0.01** | 29,83 | 1.37 | **<.01** |
| Age (1998-2012) |  |  |  | -0.02 | **<.01** |  | 0.93 | **<.01** |
|  |  |  |  |  |  |  |  |  |
| 2012 vs 1998 |  |  |  | 0.06 | **<.01** |  | 1.30 | **<.01** |
| **Women, 30-39 years** | | | | | | | | |
| 1998 | 725 | 1.35 | 0,52 |  |  | 18,48 |  |  |
| 2002 | 536 | 1.34 | 0,54 | -0.004 | 0.74 | 18,10 | 0.99 | 0.87 |
| 2005 | 509 | 1.32 | 0,50 | -0.01 | 0.52 | 17,29 | 0.98 | 0.76 |
| 2008 | 397 | 1.35 | 0,47 | 0.01 | 0.49 | 18,14 | 1.03 | 0.70 |
| 2012 | 273 | 1.39 | 0,55 | 0.02 | 0.32 | 22,71 | 1.13 | 0.19 |
| Age (1998-2012) |  |  |  | 0.003 | 0.38 |  | 1.01 | 0.43 |
|  |  |  |  |  |  |  |  |  |
| 2012 vs 1998 |  |  |  | 0.01 | 0.66 |  | 1.09 | 0.33 |
|  |  |  |  |  |  |  |  |  |
| **Women, 40-49 years** | | | | | | | | |
| 1998 | 658 | 1.36 | 0.50 |  |  | 19,00 |  |  |
| 2002 | 527 | 1.34 | 0.50 | -0.009 | 0.50 | 18,22 | 0.97 | 0.67 |
| 2005 | 494 | 1.32 | 0.48 | -0.01 | 0.47 | 17,21 | 0.97 | 0.69 |
| 2008 | 443 | 1.37 | 0.53 | 0.03 | 0.10 | 17,38 | 1.01 | 0.92 |
| 2012 | 335 | 1.37 | 0.52 | -0.003 | 0.83 | 17,91 | 1.02 | 0.82 |
| Age (1998-2012) |  |  |  | -0.002 | 0.48 |  | 1.01 | 0.51 |
|  |  |  |  |  |  |  |  |  |
| 2012 vs 1998 |  |  |  | 0.001 | 0.96 |  | 0.95 | 0.56 |
|  |  |  |  |  |  |  |  |  |
| **Women, 50-59 years** | | | | | | | | |
| 1998 | 541 | 1.41 | 0.58 |  |  | 22,00 |  |  |
| 2002 | 492 | 1.36 | 0.54 | -0.02 | 0.15 | 17,48 | 0.87 | 0.07 |
| 2005 | 436 | 1.35 | 0.52 | -0.003 | 0.85 | 19,72 | 1.08 | 0.37 |
| 2008 | 449 | 1.37 | 0.51 | 0.01 | 0.70 | 20,71 | 1.03 | 0.71 |
| 2012 | 397 | 1.33 | 0.49 | -0.02 | 0.29 | 17,13 | 0.89 | 0.20 |
| Age (1998-2012) |  |  |  | -0.01 | 0.28 |  |  |  |
|  |  |  |  |  |  |  |  |  |
| 2012 vs 1998 |  |  |  | -0.04 | **0.02** |  | 0.86 | 0.06 |
|  |  |  |  |  |  |  |  |  |
| **Women, 60-69 years** | | | | | | | | |
| 1998 | 370 | 1.42 | 0.55 |  |  | 23,51 |  |  |
| 2002 | 327 | 1.35 | 0.47 | -0.04 | **.03** | 18,96 | 0.82 | **0.06** |
| 2005 | 342 | 1.24 | 0.39 | -0.05 | **<.01** | 11,11 | 0.75 | **<.01** |
| 2008 | 368 | 1.28 | 0.41 | 0.02 | 0.30 | 13,86 | 1.09 | 0.39 |
| 2012 | 375 | 1.29 | 0.45 | 0.006 | 0.71 | 14,40 | 1.03 | 0.80 |
| Age (1998-2012) |  |  |  |  |  |  |  |  |
|  |  |  |  |  |  |  |  |  |
| 2012 vs 1998 |  |  |  | -0.06 | **<.01** |  | 0.71 | **<.01** |
|  |  |  |  |  |  |  |  |  |
| **Women, 70-79 years** | | | | | | | | |
| **Year** | **N=** | **m=** | **sd=** | **Reg. Coefficient comparing this year to the year prior** | **P=** | **Percentage scoring above ≥1.80** | **OR for being above cut of in this year compared to the year prior** | **P=** |
| 1998 | 317 | 1.44 | 0.56 |  |  | 23,34 |  |  |
| 2002 | 218 | 1.42 | 0.55 | -0.02 | 0.35 | 22,02 | 0.92 | 0.44 |
| 2005 | 168 | 1.31 | 0.43 | -0.05 | **0.04** | 18,45 | 0.89 | 0.38 |
| 2008 | 185 | 1.32 | 0.42 | 0.001 | 0.97 | 14,05 | 0.85 | 0.22 |
| 2012 | 239 | 1.32 | 0.43 | -0.01 | 0.74 | 15,48 | 1.02 | 0.87 |
| Age (1998-2012) |  |  |  |  |  |  |  |  |
|  |  |  |  |  |  |  |  |  |
| 2012 vs 1998 |  |  |  | -0.06 | **<.01** |  | 0.78 | **0.02** |
|  |  |  |  |  |  |  |  |  |

*The Living Conditions Survey (paper version) also included participants aged 80-89 years old. However, we only include data for participants aged 80-89 for surveys that had close to n>100 for each time point. The Living Conditions Survey (paper version) did not fulfill this criteria

**Table 1b.** Mean score and percentage scoring above cut-off level for mental health problems by age group for men in the Living Conditions Survey (paper version).

| **Living Conditions Survey (paper version)** | | | | | | | | |
| --- | --- | --- | --- | --- | --- | --- | --- | --- |
| **Year** | **N=** | **m=** | **sd=** | **Reg. Coefficient comparing this year to the year prior** | **P=** | **Percentage scoring ≥2.00** | **OR for being above cut of in this year compared to the year prior** | **P=** |
| **Men, 20-29 years** | | | | | | | | |
| 1998 | 486 | 1.35 | 0.53 |  |  | 11.73 |  |  |
| 2002 | 325 | 1.33 | 0.51 | -0.01 | 0.59 | 13.23 | 1.06 | 0.59 |
| 2005 | 284 | 1.29 | 0.50 | -0.02 | 0.42 | 10.21 | 0.86 | 0.25 |
| 2008 | 219 | 1.35 | 0.50 | 0.03 | 0.19 | 9.59 | 0.99 | 0.96 |
| 2012 | 197 | 1.38 | 0.58 | 0.01 | 0.59 | 13.71 | 1.22 | 0.19 |
| Age (1998-2012) |  |  |  | -0.01 | 0.55 |  | 0.99 | 0.93 |
|  |  |  |  |  |  |  |  |  |
| 2012 vs 1998 |  |  |  | 0.01 | 0.86 |  | 1.06 | 0.63 |
| **Men, 30-39 years** | | | | | | | | |
| 1998 | 680 | 1.28 | 0.45 |  |  | 9.12 |  |  |
| 2002 | 483 | 1.28 | 0.45 | 0.001 | 0.91 | 7.66 | 0.94 | 0.56 |
| 2005 | 406 | 1.28 | 0.46 | -0.00 | 0.99 | 9.61 | 1.13 | 0.28 |
| 2008 | 297 | 1.33 | 0.51 | 0.02 | 0.17 | 12.79 | 1.17 | 0.22 |
| 2012 | 228 | 1.27 | 0.40 | 0.03 | 0.11 | 9.65 | 0.80 | 0.15 |
| Age (1998-2012) |  |  |  | 0.01 | 0.85 |  | 0.97 | 0.21 |
|  |  |  |  |  |  |  |  |  |
| 2012 vs 1998 |  |  |  | 0.01 | 0.92 |  | 1.05 | 0.67 |
|  |  |  |  |  |  |  |  |  |
| **Men, 40-49 years** | | | | | | | | |
| **Year** | **N=** | **m=** | **sd=** | **Reg. Coefficient comparing this year to the year prior** | **P=** | **Percentage scoring ≥2.00** | **OR for being above cut of in this year compared to the year prior** | **P=** |
| 1998 | 613 | 1.32 | 0.48 |  |  | 10.60 |  |  |
| 2002 | 544 | 1.27 | 0.47 | -0.03 | 0.08 | 9.01 | 0.91 | 0.35 |
| 2005 | 431 | 1.24 | 0.45 | -0.01 | 0.47 | 7.89 | 0.96 | 0.67 |
| 2008 | 417 | 1.32 | 0.51 | 0.04 | **0.02** | 10.31 | 1.15 | 0.21 |
| 2012 | 312 | 1.32 | 0.48 | -0.01 | 0.75 | 11.86 | 1.06 | 0.64 |
| Age (1998-2012) |  |  |  | 0.01 | 0.49 |  | 1.04 | 0.14 |
|  |  |  |  |  |  |  |  |  |
| 2012 vs 1998 |  |  |  | -0.01 | 0.50 |  | 1.01 | 0.90 |
|  |  |  |  |  |  |  |  |  |
| **Men, 50-59 years** | | | | | | | | |
| 1998 | 523 | 1.31 | 0.45 |  |  | 11.28 |  |  |
| 2002 | 490 | 1.29 | 0.47 | -0.01 | 0.53 | 11.22 | 0.99 | 0.97 |
| 2005 | 444 | 1.28 | 0.46 | -0.01 | 0.64 | 9.23 | 0.91 | 0.35 |
| 2008 | 388 | 1.29 | 0.44 | 0.01 | 0.65 | 9.54 | 1.02 | 0.82 |
| 2012 | 381 | 1.32 | 0.47 | 0.02 | 0.36 | 13.39 | 1.22 | 0.09 |
| Age (1998-2012) |  |  |  | 0.001 | 0.84 |  | 0.99 | 0.82 |
|  |  |  |  |  |  |  |  |  |
| 2012 vs 1998 |  |  |  | 0.002 | 0.89 |  | 1.09 | 0.41 |
|  |  |  |  |  |  |  |  |  |
| **Men, 60-69 years** | | | | | | | | |
| 1998 | 373 | 1.26 | 0.44 |  |  | 8.85 |  |  |
| 2002 | 335 | 1.26 | 0.42 | -0.003 | 0.83 | 8.06 | 0.93 | 0.60 |
| 2005 | 348 | 1.19 | 0.39 | -0.04 | **0.02** | 5.17 | 0.80 | 0.13 |
| 2008 | 365 | 1.24 | 0.40 | 0.03 | 0.11 | 8.49 | 1.26 | 0.11 |
| 2012 | 377 | 1.26 | 0.45 | 0.01 | 0.37 | 7.16 | 0.93 | 0.59 |
| Age (1998-2012) |  |  |  | -0.01 | 0.04 |  | 0.93 | **0.02** |
|  |  |  |  |  |  |  |  |  |
| 2012 vs 1998 |  |  |  | 0.0001 | 0.96 |  | 0.88 | 0.37 |
|  |  |  |  |  |  |  |  |  |
| **Men, 70-79 years** | | | | | | | | |
| 1998 | 270 | 1.27 | 0.40 |  |  | 10.37 |  |  |
| 2002 | 202 | 1.25 | 0.48 | -0.02 | 0.36 | 9.41 | 0.88 | 0.48 |
| 2005 | 193 | 1.17 | 0.33 | -0.04 | 0.06 | 4.66 | 0.71 | 0.07 |
| 2008 | 166 | 1.17 | 0.29 | 0.001 | 0.94 | 4.22 | 1.01 | 0.98 |
| 2012 | 183 | 1.23 | 0.36 | 0.03 | 0.19 | 6.01 | 1.11 | 0.59 |
| Age (1998-2012) |  |  |  | 0.001 | 0.71 |  |  |  |
|  |  |  |  |  |  |  |  |  |
| 2012 vs 1998 |  |  |  | -0.03 | 0.14 |  | 0.70 | **0.056** |
|  |  |  |  |  |  |  |  |  |

*The Living Conditions Survey (paper version) also included participants aged 80-89 years old. However, we only include data for participants aged 80-89 for surveys that had close to n>100 for each time point. The Living Conditions Survey (paper version) did not fulfill these criteria

**Table 2a.** Mean score and percentage scoring above cut-off level for mental health problems by age group for women in the Living Conditions Survey (telephone version)

| **Living Conditions Survey (telephone version)** | | | | | | | | |
| --- | --- | --- | --- | --- | --- | --- | --- | --- |
| **Year** | **N=** | **m=** | **sd=** | **Reg. Coefficient comparing this year to the year prior** | **P=** | **Percentage scoring ≥ 1.80** | **OR for being above cut of in this year compared to the year prior** | **P=** |
| **Women, 20-29 years** | | | | | | | | |
| 2015 | 585 | 1.36 | 0.54 |  |  | 17.61 |  |  |
| 2019 | 550 | 1.50 | 0.61 | 0.07 | **<.01** | 25.64 | 1.27 | **<.01** |
| Age |  |  |  | -0.02 | 0.01 |  | 0.93 | 0.01 |
|  |  |  |  |  |  |  |  |  |
| **Women 30-39 years old** | | | | | | | | |
| 2015 | 561 | 1.30 | 0.48 |  |  | 15.15 |  |  |
| 2019 | 621 | 1.40 | 0.55 | 0.05 | **<.01** | 20.00 | 1.18 | **0.03** |
| Age |  |  |  | -0.01 | 0.57 |  | 0.99 | 0.71 |
|  |  |  |  |  |  |  |  |  |
| **Women 40-49 years old** | | | | | | | | |
| 2015 | 714 | 1.29 | 0.47 |  |  | 13.45 |  |  |
| 2019 | 643 | 1.33 | 0.50 | 0.02 | 0.11 | 14.93 | 1.06 | 0.43 |
| Age |  |  |  | 0.02 | 0.11 |  | 1.00 | 0.99 |
|  |  |  |  |  |  |  |  |  |
| **Women 50-59 years old** | | | | | | | | |
| 2015 | 685 | 1.22 | 0.40 |  |  | 10.66 |  |  |
| 2019 | 675 | 1.35 | 0.54 | 0.06 | **<.01** | 18.07 | 1.36 | **<.01** |
| Age |  |  |  | 0.01 | 0.78 |  | 1.02 | 0.43 |
|  |  |  |  |  |  |  |  |  |
| **Women 60-69 years old** | | | | | | | | |
| 2015 | 637 | 1.19 | 0.39 |  |  | 9.58 |  |  |
| 2019 | 587 | 1.23 | 0.40 | 0.02 | **0.05** | 11.24 | 1.09 | 0.34 |
| Age |  |  |  | -0.01 | 0.34 |  | 0.98 | 0.64 |
|  |  |  |  |  |  |  |  |  |
| **Women 70-79 years old** | | | | | | | | |
| 2015 | 341 | 1.17 | 0.35 |  |  | 7.92 |  |  |
| 2019 | 411 | 1.23 | 0.38 | 0.03 | **0.05** | 11.44 | 1.22 | 0.10 |
| Age |  |  |  | -0.01 | 0.81 |  | 0.97 | 0.57 |
|  |  |  |  |  |  |  |  |  |
| **Women 80-89 years old** | | | | | | | | |
| 2015 | 177 | 1.19 | 0.41 |  |  | 6.21 |  |  |
| 2019 | 183 | 1.26 | 0.42 | 0.04 | 0.09 | 10.93 | 1.35 | 0.12 |
| Age |  |  |  | 0.01 | 0.22 |  | 1.10 | 0.16 |

**Table 2b.** Mean score and percentage scoring above cut-off level for mental health problems by age group for men in the Living Conditions Survey (telephone version)

| **Living Conditions Survey (telephone version)** | | | | | | | | |
| --- | --- | --- | --- | --- | --- | --- | --- | --- |
| **Year** | **N=** | **m=** | **sd=** | **Reg. Coefficient comparing this year to the year prior** | **P=** | **Percentage scoring ≥2.00** | **OR for being above cut of in this year compared to the year prior** | **P=** |
| **Men, 20-29 years** | | | | | | | | |
| 2015 | 600 | 1.23 | 0.39 |  |  | 6.17 |  |  |
| 2019 | 589 | 1.30 | 0.47 | 0.04 | **<.01** | 10.53 | 1.34 | **0.01** |
| Age |  |  |  | -0.01 | .42 |  | 0.97 | 0.39 |
|  |  |  |  |  |  |  |  |  |
| **Men 30-39 years old** | | | | | | | | |
| 2015 | 586 | 1.22 | 0.44 |  |  | 7.68 |  |  |
| 2019 | 566 | 1.26 | 0.45 | 0.02 | 0.13 | 8.66 | 1.07 | 0.52 |
| Age |  |  |  | 0.01 | 0.15 |  | 1.05 | 0.16 |
|  |  |  |  |  |  |  |  |  |
| **Men 40-49 years old** | | | | | | | | |
| 2015 | 710 | 1.17 | 0.39 |  |  | 5.07 |  |  |
| 2019 | 654 | 1.22 | 0.42 | 0.03 | **0.01** | 7.19 | 1.20 | 0.12 |
| Age |  |  |  | 0.01 | 0.57 |  | 1.04 | 0.23 |
|  |  |  |  |  |  |  |  |  |
| **Men 50-59 years old** | | | | | | | | |
| 2015 | 688 | 1.13 | 0.35 |  |  | 3.78 |  |  |
| 2019 | 701 | 1.21 | 0.42 | 0.04 | **<.01** | 5.56 | 1.22 | 0.12 |
| Age |  |  |  | 0.01 | 0.69 |  | 0.99 | 0.98 |
|  |  |  |  |  |  |  |  |  |
| **Men 60-69 years old** | | | | | | | | |
| 2015 | 687 | 1.11 | 0.29 |  |  | 2.47 |  |  |
| 2019 | 628 | 1.15 | 0.33 | 0.02 | **0.02** | 4.30 | 1.32 | 0.08 |
| Age |  |  |  | -0.01 | 0.03 |  | 0.89 | 0.30 |
|  |  |  |  |  |  |  |  |  |
| **Men 70-79 years old** | | | | | | | | |
| 2015 | 368 | 1.07 | 0.21 |  |  | 2.17 |  |  |
| 2019 | 432 | 1.11 | 0.25 | 0.02 | **0.03** | 2.55 | 1.08 | 0.74 |
| Age |  |  |  | 0.01 | 0.39 |  | 1.04 | 0.61 |
|  |  |  |  |  |  |  |  |  |
| **Men 80-89 years old** | | | | | | | | |
| 2015 | 131 | 1.13 | 0.29 |  |  | 3.05 |  |  |
| 2019 | 135 | 1.17 | 0.37 | 0.02 | 0.31 | 5.19 | 1.34 | 0.36 |
| Age |  |  |  | 0.01 | 0.15 |  | 1.13 | 0.28 |

**Table 3a.** Mean score and percentage scoring above cut-off level for mental health problems by age group for women in HUNT

| **HUNT** | | | | | | | | |
| --- | --- | --- | --- | --- | --- | --- | --- | --- |
|  |  | **Anxiety**  **HADS-A mean score** | | | | **Anxiety**  **HADS- A cut off** | | |
| **Year** | **N=** | **m=** | **SD=** | **Reg. Coefficient comparing this year to the year prior** | **P=** | **Percentage scoring ≥8** | **OR for being above cut of in this year compared to the year prior** | **P=** |
| **Women, 20-29 years** | | | | | | | | |
| 1995-97 | 4655 | 4.44 | 3.19 |  |  | 15.45 |  |  |
| 2006-08 | 1796 | 4.84 | 3.34 | 0.40 | **<.01** | 19.10 | 1.25 | **<.01** |
| 2017-19 | 2399 | 6.08 | 3.95 | 0.70 | **<.01** | 32.00 | 1.57 | **<.01** |
|  |  |  |  |  |  |  |  |  |
| Age (1995 – 2017) |  |  |  | -0.04 | **<.01** |  | 0.99 | 0.70 |
|  |  |  |  |  |  |  |  |  |
| 1995-17 vs 2017-19 |  |  |  | 0.79 | **<.01** |  | 1.60 | **<.01** |
| **Women, 30-39 years** | | | | | | | | |
| 1995-97 | 5766 | 4.48 | 3.41 |  |  | 17.07 |  |  |
| 2006-08 | 3036 | 4.37 | 3.39 | 0.03 | 0.41 | 17.79 | 1.07 | **.01** |
| 2017-19 | 2733 | 5.45 | 3.67 | 0.52 | **<.01** | 26.67 | 1.31 | **<.01** |
|  |  |  |  |  |  |  |  |  |
| Age (1995 – 2017) |  |  |  | -0.02 | 0.04 |  | 0.98 | 0.15 |
|  |  |  |  |  |  |  |  |  |
| 1995-17 vs 2017-19 |  |  |  | 0.42 | **<.01** |  | 1.31 | **<.01** |
| **Women, 40-49 years** | | | | | | | | |
| **Year** | **N=** | **m=** | **sd=** | **Reg. Coefficient comparing this year to the year prior** | **P=** | **Percentage scoring ≥8** | **OR for being above cut of in this year compared to the year prior** | **P=** |
| 1995-97 | 6300 | 4.53 | 3.48 |  |  | 17.89 |  |  |
| 2006-08 | 4393 | 4.36 | 3.54 | -0.06 | 0.06 | 17.05 | 0.98 | 0.58 |
| 2017-19 | 3844 | 4.85 | 3.67 | 0.24 | **<.01** | 22.09 | 1.17 | **<.01** |
|  |  |  |  |  |  |  |  |  |
| Age (1995 – 2017) |  |  |  | 0.01 | 0.31 |  | 1.01 | 0.15 |
|  |  |  |  |  |  |  |  |  |
| 1995-17 vs 2017-19 |  |  |  | 0.13 | **<.01** |  | 1.13 | **<.01** |
| **Women, 50-59 years** | | | | | | | | |
| 1995-97 | 4571 | 4.59 | 3.54 |  |  | 18.64 |  |  |
| 2006-08 | 4919 | 4.36 | 3.56 | -0.12 | **<.01** | 18.01 | 0.97 | 0.37 |
| 2017-19 | 4718 | 4.62 | 3.59 | 0.13 | **<.01** | 20.41 | 1.08 | **<.01** |
|  |  |  |  |  |  |  |  |  |
| Age (1995 – 2017) |  |  |  | 0.01 | 0.40 |  | 1.001 | 0.59 |
|  |  |  |  |  |  |  |  |  |
| 1995-17 vs 2017-19 |  |  |  | 0.02 | 0.67 |  | 1.05 | **0.03** |
| **Women, 60-69 years** | | | | | | | | |
| 1995-97 | 3229 | 4.52 | 3.6 |  |  | 17.96 |  |  |
| 2006-08 | 4362 | 4.28 | 3.41 | -0.12 | **<.01** | 16.41 | 0.94 | 0.06 |
| 2017-19 | 4838 | 4.39 | 3.48 | 0.05 | 0.14 | 17.86 | 1.05 | 0.07 |
|  |  |  |  |  |  |  |  |  |
| Age (1995 – 2017) |  |  |  | -0.003 | 0.78 |  | 0.99 | 0.63 |
|  |  |  |  |  |  |  |  |  |
| 1995-17 vs 2017-19 |  |  |  | -0.05 | 0.21 |  | 1.00 | 0.89 |
| **Women, 70-79 years** | | | | | | | | |
| 1995-97 | 2290 | 4.24 | 3.49 |  |  | 17.16 |  |  |
| 2006-08 | 2475 | 4.41 | 3.35 | 0.08 | 0.09 | 17.21 | 1.00 | 0.94 |
| 2017-19 | 3535 | 4.25 | 3.23 | -0.08 | 0.07 | 16.18 | 0.96 | 0.27 |
|  |  |  |  |  |  |  |  |  |
| Age (1995 – 2017) |  |  |  | -0.02 | 0.05 |  | 0.99 | 0.81 |
|  |  |  |  |  |  |  |  |  |
| 1995-17 vs 2017-19 |  |  |  | -0.01 | 0.97 |  | 0.96 | 0.27 |
|  |  |  |  |  |  |  |  |  |
| **Women, 80+ years** |  |  |  |  |  |  |  |  |
| 1995-97 | 763 | 4.19 | 3.59 |  |  | 18.35 |  |  |
| 2006-08 | 891 | 3.97 | 3.31 | -0.13 | 0.13 | 14.14 | 0.85 | **0.02** |
| 2017-19 | 1111 | 4.54 | 3.34 | 0.30 | **<.01** | 18.27 | 1.17 | **0.01** |
|  |  |  |  |  |  |  |  |  |
| Age (1995 – 2017) |  |  |  | -0.08 | <.01 |  | 0.95 | <.01 |
|  |  |  |  |  |  |  |  |  |
| 1995-17 vs 2017-19 |  |  |  | 0.21 | **<.01** |  | 1.02 | 0.71 |

| **HUNT** | | | | | | | | |
| --- | --- | --- | --- | --- | --- | --- | --- | --- |
|  |  | **Depression**  **HADS-D mean score** | | | | **Depression**  **HADS-D cut off** | | |
| **Year** | **N=** | **m=** | **SD=** | **Reg. Coefficient comparing this year to the year prior** | **P=** | **Percentage scoring ≥8** | **OR for being above cut of in this year compared to the year prior** | **P=** |
| **Women, 20-29 years** | | | | | | | | |
| 1995-97 | 4764 | 2.25 | 2.40 |  |  | 4.22 |  |  |
| 2006-08 | 1790 | 2.26 | 2.40 | 0.22 | **<.01** | 4.58 | 1.20 | **<.01** |
| 2017-19 | 2392 | 3.31 | 3.12 | 0.57 | **<.01** | 10.70 | 1.84 | **<.01** |
|  |  |  |  |  |  |  |  |  |
| Age (1995 – 2017) |  |  |  | 0.02 | **0.03** |  | 1.02 | 0.28 |
|  |  |  |  |  |  |  |  |  |
| 1995-17 vs 2017-19 |  |  |  | 0.55 | **<.01** |  | 1.66 | **<.01** |
| **Women, 30-39 years** | | | | | | | | |
| 1995-97 | 5914 | 2.73 | 2.76 |  |  | 6.90 |  |  |
| 2006-08 | 3023 | 2.53 | 2.68 | -0.07 | **0.02** | 6.29 | 0.98 | 0.58 |
| 2017-19 | 2732 | 3.04 | 2.95 | 0.26 | **<.01** | 8.93 | 1.22 | **<.01** |
|  |  |  |  |  |  |  |  |  |
| Age (1995 – 2017) |  |  |  | 0.02 | <.01 |  | 1.03 | <.01 |
|  |  |  |  |  |  |  |  |  |
| 1995-17 vs 2017-19 |  |  |  | 0.12 | **<.01** |  | 1.13 | **<.01** |
| **Women, 40-49 years** | | | | | | | | |
| **Year** | **N=** | **m=** | **sd=** | **Reg. Coefficient comparing this year to the year prior** | **P=** | **Percentage scoring ≥8** | **OR for being above cut of in this year compared to the year prior** | **P=** |
| 1995-97 | 6691 | 3.22 | 2.95 |  |  | 9.30 |  |  |
| 2006-08 | 4381 | 2.79 | 2.83 | -0.22 | <.01 | 7.69 | 0.91 | <.01 |
| 2017-19 | 3848 | 2.92 | 2.97 | 0.07 | **0.03** | 9.04 | 1.09 | **0.03** |
|  |  |  |  |  |  |  |  |  |
| Age (1995 – 2017) |  |  |  | 0.04 | <.01 |  | 1.01 | 0.09 |
|  |  |  |  |  |  |  |  |  |
| 1995-17 vs 2017-19 |  |  |  | -0.18 | **<.01** |  | 0.97 | 0.33 |
| **Women, 50-59 years** | | | | | | | | |
| 1995-97 | 4562 | 3.74 | 3.10 |  |  | 12.33 |  |  |
| 2006-08 | 4922 | 3.18 | 2.91 | -0.29 | **<.01** | 9.02 | 0.82 | **<.01** |
| 2017-19 | 4709 | 2.94 | 2.94 | -0.11 | **<.01** | 8.43 | 0.97 | 0.39 |
|  |  |  |  |  |  |  |  |  |
| Age (1995 – 2017) |  |  |  | 0.05 | <.01 |  | 1.02 | 0.05 |
|  |  |  |  |  |  |  |  |  |
| 1995-17 vs 2017-19 |  |  |  | -0.42 | **<.01** |  | 0.80 | **<.01** |
| **Women, 60-69 years** | | | | | | | | |
| 1995-97 | 4019 | 4.08 | 3.16 |  |  | 14.21 |  |  |
| 2006-08 | 4393 | 3.41 | 2.77 | -0.31 | **<.01** | 8.81 | 0.75 | **<.01** |
| 2017-19 | 4865 | 2.99 | 2.76 | -0.23 | **<.01** | 7.42 | 0.91 | **<.01** |
|  |  |  |  |  |  |  |  |  |
| Age (1995 – 2017) |  |  |  | 0.03 | <.01 |  | 1.02 | 0.13 |
|  |  |  |  |  |  |  |  |  |
| 1995-17 vs 2017-19 |  |  |  | -0.54 | **<.01** |  | 0.77 | **<.01** |
| **Women, 70-79 years** | | | | | | | | |
| **Year** | **N=** | **m=** | **sd=** | **Reg. Coefficient comparing this year to the year prior** | **P=** | **Percentage scoring ≥8** | **OR for being above cut of in this year compared to the year prior** | **P=** |
| 1995-97 | 3199 | 4.39 | 3.29 |  |  | 17.54 |  |  |
| 2006-08 | 2569 | 4.01 | 2.90 | -0.23 | **<.01** | 12.61 | 0.77 | **<.01** |
| 2017-19 | 3614 | 3.27 | 2.70 | -0.40 | **<.01** | 7.58 | 0.77 | **<.01** |
|  |  |  |  |  |  |  |  |  |
| Age (1995 – 2017) |  |  |  | 0.05 | <.01 |  | 1.02 | 0.02 |
|  |  |  |  |  |  |  |  |  |
| 1995-17 vs 2017-19 |  |  |  | -0.55 | **<.01** |  | 0.63 | **<.01** |
|  |  |  |  |  |  |  |  |  |
| **Women, 80-89 years** | | | | | | | | |
| 1995-97 | 1042 | 4.78 | 3.53 |  |  | 19.96 |  |  |
| 2006-08 | 971 | 4.33 | 3.12 | **-**0.22 | **<.01** | 15.45 | **0.85** | **<.01** |
| 2017-19 | 1181 | 4.38 | 3.09 | -0.07 | 0.92 | 15.41 | 0.98 | 0.67 |
|  |  |  |  |  |  |  |  |  |
| Age (1995 – 2017) |  |  |  | 0.08 | <.01 |  | 1.06 | <.01 |
|  |  |  |  |  |  |  |  |  |
| 1995-17 vs 2017-19 |  |  |  | -0.21 | **<.01** |  | 0.84 | **<.01** |

**Table 3b.** Mean score and percentage scoring above cut-off level for mental health problems by age group for men in HUNT

| **HUNT** | | | | | | | | |
| --- | --- | --- | --- | --- | --- | --- | --- | --- |
|  |  | **Anxiety**  **HADS-A mean score** | | | | **Anxiety**  **HADS-A cut off** | | |
| **Year** | **N=** | **m=** | **SD=** | **Reg. Coefficient comparing this year to the year prior** | **P=** | **Percentage scoring ≥8** | **OR for being above cut of in this year compared to the year prior** | **P=** |
| **Men, 20-29 years** | | | | | | | | |
| 1995-97 | 3953 | 4.05 | 2.92 |  |  | 11.92 |  |  |
| 2006-08 | 1015 | 4.09 | 2.93 | 0.17 | **<.01** | 12.02 | 1.12 | **0.02** |
| 2017-19 | 1356 | 4.92 | 3.34 | 0.45 | **<.01** | 19.03 | 1.39 | **<.01** |
|  |  |  |  |  |  |  |  |  |
| Age (1995 – 2017) |  |  |  | 0.04 | <.01 |  | 1.04 | <.01 |
|  |  |  |  |  |  |  |  |  |
| 1995-17 vs 2017-19 |  |  |  | 0.39 | **<.01** |  | 1.30 | **<.01** |
| **Men, 30-39 years** | | | | | | | | |
| 1995-97 | 5119 | 4.14 | 3.11 |  |  | 12.93 |  |  |
| 2006-08 | 1847 | 3.85 | 3.00 | -0.05 | 0.24 | 11.37 | 1.01 | 0.96 |
| 2017-19 | 1696 | 4.76 | 3.31 | 0.44 | **<.01** | 18.81 | 1.36 | **<.01** |
|  |  |  |  |  |  |  |  |  |
| Age (1995 – 2017) |  |  |  | -0.04 | <.01 |  | 0.98 | 0.09 |
|  |  |  |  |  |  |  |  |  |
| 1995-17 vs 2017-19 |  |  |  | 0.23 | **<.01** |  | 1.21 | **<.01** |
| **Men, 40-49 years** | | | | | | | | |
| **Year** | **N=** | **m=** | **sd=** | **Reg. Coefficient comparing this year to the year prior** | **P=** | **Percentage scoring ≥8** | **OR for being above cut of in this year compared to the year prior** | **P=** |
| 1995-97 | 5989 | 4.07 | 3.22 |  |  | 14.04 |  |  |
| 2006-08 | 3328 | 3.86 | 3.11 | -0.08 | **0.02** | 12.50 | 0.95 | 0.14 |
| 2017-19 | 2506 | 4.30 | 3.30 | 0.22 | **<.01** | 16.52 | 1.18 | **<.01** |
|  |  |  |  |  |  |  |  |  |
| Age (1995 – 2017) |  |  |  | -0.02 | 0.03 |  | 0.98 | 0.16 |
|  |  |  |  |  |  |  |  |  |
| 1995-17 vs 2017-19 |  |  |  | 0.08 | **0.04** |  | 1.07 | **0.03** |
| **Men, 50-59 years** | | | | | | | | |
| 1995-97 | 4655 | 3.88 | 3.15 |  |  | 12.50 |  |  |
| 2006-08 | 4294 | 3.66 | 3.18 | -0.11 | **<.01** | 11.74 | 0.97 | 0.36 |
| 2017-19 | 3479 | 3.99 | 3.27 | 0.17 | **<.01** | 15.18 | 1.16 | **<.01** |
|  |  |  |  |  |  |  |  |  |
| Age (1995 – 2017) |  |  |  | -0.01 | 0.33 |  | 0.99 | 0.35 |
|  |  |  |  |  |  |  |  |  |
| 1995-17 vs 2017-19 |  |  |  | 0.04 | 0.20 |  | 1.12 | **<.01** |
| **Men, 60-69 years** | | | | | | | | |
| 1995-97 | 3351 | 3.37 | 2.91 |  |  | 9.19 |  |  |
| 2006-08 | 3924 | 3.23 | 2.88 | -0.09 | **0.01** | 8.46 | 0.95 | 0.17 |
| 2017-19 | 4243 | 3.48 | 3.01 | 0.14 | <.01 | 11.03 | 1.17 | **<.01** |
|  |  |  |  |  |  |  |  |  |
| Age (1995 – 2017) |  |  |  | -0.05 | <.01 |  | 0.96 | <.01 |
|  |  |  |  |  |  |  |  |  |
| 1995-17 vs 2017-19 |  |  |  | 0.07 | **0.04** |  | 1.13 | **<.01** |
| **Men, 70-79 years** | | | | | | | | |
| 1995-97 | 2321 | 3.32 | 2.92 |  |  | 9.35 |  |  |
| 2006-08 | 2195 | 3.16 | 2.63 | -0.08 | **0.05** | 6.56 | 0.83 | **<.01** |
| 2017-19 | 3251 | 3.14 | 2.80 | -0.02 | 0.65 | 8.40 | 1.12 | **0.03** |
|  |  |  |  |  |  |  |  |  |
| Age (1995 – 2017) |  |  |  | 0.01 | 0.35 |  | 1.01 | 0.62 |
|  |  |  |  |  |  |  |  |  |
| 1995-17 vs 2017-19 |  |  |  | -0.08 | **0.03** |  | 0.95 | 0.33 |
|  |  |  |  |  |  |  |  |  |
| **Men, 80-89 years** |  |  |  |  |  |  |  |  |
| 1995-97 | 538 | 3.19 | 2.97 |  |  | 10.59 |  |  |
| 2006-08 | 703 | 3.20 | 2.78 | -0.01 | 0.93 | 8.82 | 0.90 | 0.29 |
| 2017-19 | 934 | 3.41 | 3.04 | 0.11 | 0.13 | 9.85 | 1.05 | 0.54 |
|  |  |  |  |  |  |  |  |  |
| Age (1995 – 2017) |  |  |  | 0.01 | 0.97 |  | 1.01 | 0.78 |
|  |  |  |  |  |  |  |  |  |
| 1995-17 vs 2017-19 |  |  |  | 0.12 | 0.13 |  | 0.97 | 0.75 |

| **HUNT** | | | | | | | | |
| --- | --- | --- | --- | --- | --- | --- | --- | --- |
|  |  | **Depression**  **HADS-D mean score** | | | | **Depression**  **HADS-D cut off** | | |
| **Year** | **N=** | **Mean score=** | **SD=** | **Reg. Coefficient comparing this year to the year prior** | **P=** | **Percentage scoring ≥8** | **OR for being above cut of in this year compared to the year prior** | **P=** |
| **Men, 20-29 years** | | | | | | | | |
| 1995-97 | 3984 | 2.34 | 2.35 |  |  | 3.89 |  |  |
| 2006-08 | 1016 | 2.57 | 2.59 | 0.29 | **<.01** | 5.81 | 1.39 | **<.01** |
| 2017-19 | 1353 | 3.47 | 3.01 | 0.51 | **<.01** | 10.20 | 1.64 | **<.01** |
|  |  |  |  |  |  |  |  |  |
| Age (1995 – 2017) |  |  |  | 0.03 | <.01 |  | 1.02 | 0.23 |
|  |  |  |  |  |  |  |  |  |
| 1995-17 vs 2017-19 |  |  |  | 0.53 | **<.01** |  | 1.68 | **<.01** |
| **Men, 30-39 years** | | | | | | | | |
| 1995-97 | 5225 | 2.92 | 2.69 |  |  | 6.85 |  |  |
| 2006-08 | 1839 | 2.88 | 2.70 | 0.05 | 0.15 | 7.29 | 1.09 | **0.05** |
| 2017-19 | 1697 | 3.64 | 3.06 | 0.39 | **<.01** | 11.61 | 1.34 | **<.01** |
|  |  |  |  |  |  |  |  |  |
| Age (1995 – 2017) |  |  |  | 0.04 | <.01 |  | 1.01 | 0.29 |
|  |  |  |  |  |  |  |  |  |
| 1995-17 vs 2017-19 |  |  |  | 0.30 | **<.01** |  | 1.31 | **<.01** |
| **Men, 40-49 years** | | | | | | | | |
| **Year** | **N=** | **m=** | **sd=** | **Reg. Coefficient comparing this year to the year prior** | **P=** | **Percentage scoring ≥8** | **OR for being above cut of in this year compared to the year prior** | **P=** |
| 1995-97 | 6209 | 3.58 | 3.03 |  |  | 10.44 |  |  |
| 2006-08 | 3323 | 3.24 | 2.87 | -0.17 | **<.01** | 8.97 | 0.92 | **0.03** |
| 2017-19 | 2499 | 3.42 | 3.02 | 0.10 | **.01** | 10.16 | 1.07 | 0.11 |
|  |  |  |  |  |  |  |  |  |
| Age (1995 – 2017) |  |  |  | 0.03 | <.01 |  | 1.02 | 0.02 |
|  |  |  |  |  |  |  |  |  |
| 1995-17 vs 2017-19 |  |  |  | -0.12 | **<.01** |  | 0.96 | 0.30 |
| **Men, 50-59 years** | | | | | | | | |
| 1995-97 | 4976 | 4.10 | 3.17 |  |  | 13.59 |  |  |
| 2006-08 | 4308 | 3.57 | 2.97 | -0.28 | **<.01** | 10.47 | 0.84 | **<.01** |
| 2017-19 | 3499 | 3.25 | 2.91 | -0.13 | **<.01** | 9.40 | 0.96 | 0.29 |
|  |  |  |  |  |  |  |  |  |
| Age (1995 – 2017) |  |  |  | 0.03 | <.01 |  | 1.01 | 0.32 |
|  |  |  |  |  |  |  |  |  |
| 1995-17 vs 2017-19 |  |  |  | -0.44 | **<.01** |  | 0.80 | **<.01** |
| **Men, 60-69 years** | | | | | | | | |
| 1995-97 | 3805 | 4.03 | 3.11 |  |  | 13.85 |  |  |
| 2006-08 | 3938 | 3.78 | 2.88 | -0.13 | **<.01** | 11.10 | 0.87 | **<.01** |
| 2017-19 | 4262 | 3.23 | 2.84 | -0.28 | **<.01** | 8.42 | 0.86 | **<.01** |
|  |  |  |  |  |  |  |  |  |
| Age (1995 – 2017) |  |  |  | -0.01 | 0.27 |  | 0.99 | 0.91 |
|  |  |  |  |  |  |  |  |  |
| 1995-17 vs 2017-19 |  |  |  | -0.40 | **<.01** |  | 0.75 | **<.01** |
| **Men, 70-79 years** | | | | | | | | |
| 1995-97 | 2857 | 4.36 | 3.21 |  |  | 16.77 |  |  |
| 2006-08 | 2257 | 4.23 | 2.9 | -0.11 | **0.01** | 13.74 | 0.86 | **<.01** |
| 2017-19 | 3306 | 3.6 | 2.86 | -0.33 | **<.01** | 10.53 | 0.85 | **<.01** |
|  |  |  |  |  |  |  |  |  |
| Age (1995 – 2017) |  |  |  | 0.08 | <.01 |  | 1.04 | <.01 |
|  |  |  |  |  |  |  |  |  |
| 1995-17 vs 2017-19 |  |  |  | -0.38 | **<.01** |  | 0.77 | **<.01** |
|  |  |  |  |  |  |  |  |  |
| **Men, 80-89 years** |  |  |  |  |  |  |  |  |
| 1995-97 | 719 | 5.02 | 3.49 |  |  | 23.09 |  |  |
| 2006-08 | 737 | 4.73 | 3.10 | -0.13 | 0.12 | 19.81 | 0.91 | 0.15 |
| 2017-19 | 972 | 4.73 | 3.27 | -0.03 | 0.68 | 18.83 | 0.94 | 0.35 |
|  |  |  |  |  |  |  |  |  |
| Age (1995 – 2017) |  |  |  | 0.10 | <.01 |  | 1.07 | <.01 |
|  |  |  |  |  |  |  |  |  |
| 1995-17 vs 2017-19 |  |  |  | -0.15 | 0.06 |  | 0.87 | **0.02** |

**Table 4a.** Mean score and percentage scoring above cut-off level for mental health problems by age group for women in the Quality of life survey

| **Quality of life survey** | | | | | | | | |
| --- | --- | --- | --- | --- | --- | --- | --- | --- |
| **Year** | **N=** | **m=** | **sd=** | **Reg. Coefficient comparing this year to the year prior** | **P=** | **Percentage scoring above ≥1.80** | **OR for being above cut of in this year compared to the year prior** | **P=** |
| **Women, 20-29 years** | | | | | | | | |
| 2020 | 1494 | 1.91 | 0.73 |  |  | 52,74 |  |  |
| 2021 | 1421 | 2.03 | 0.78 | 0.06 | **<.01** | 59,25 | 1.15 | **<.01** |
| 2022 | 1155 | 2.11 | 0.79 | 0.04 | **.01** | 63,03 | 1.07 | .07 |
| 2023 | 1443 | 2.07 | 0.79 | -0.01 | 0.39 | 61,19 | .98 | .59 |
| 2024 | 1338 | 2.07 | 0.76 | -0.01 | 0.81 | 61.96 | 1.01 | 0.80 |
|  |  |  |  |  |  |  |  |  |
| Age (2023- 2020) |  |  |  | -0.03 | **<.01** |  | 0.95 | <.01 |
|  |  |  |  |  |  |  |  |  |
| 2024 vs 2020 |  |  |  | 0.08 | **<.01** |  | 1.22 | **<.01** |
| **Women, 30-39 years** | | | | | | | | |
| 2020 | 1433 | 1.72 | 0.65 |  |  | 42.64 |  |  |
| 2021 | 1501 | 1.77 | 0.68 | 0.02 | 0.07 | 46.30 | 1.07 | 0.057 |
| 2022 | 1244 | 1.86 | 0.70 | 0.04 | **<.01** | 52.01 | 1.12 | **<.01** |
| 2023 | 1600 | 1.87 | 0.73 | 0.01 | 0.32 | 50.38 | 0.98 | 0.66 |
| 2024 | 1580 | 1.89 | 0.71 | 0.01 | 0.39 | 53.29 | 1.06 | 0.09 |
|  |  |  |  |  |  |  |  |  |
| Age (2023- 2020) |  |  |  | -0.02 | <.01 |  | 0.97 | <.01 |
|  |  |  |  |  |  |  |  |  |
| 2024 vs 2020 |  |  |  | 0.09 | **<.01** |  | 1.23 | **<.01** |
|  |  |  |  |  |  |  |  |  |
| **Women, 40-49 years** | | | | | | | | |
| **Year** | **N=** | **m=** | **sd=** | **Reg. Coefficient comparing this year to the year prior** | **P=** | **Percentage scoring above ≥1.80** | **OR for being above cut of in this year compared to the year prior** | **P=** |
| 2020 | 1641 | 1.60 | 0.60 |  |  | 34.67 |  |  |
| 2021 | 1594 | 1.64 | 0.63 | 0.02 | 0.06 | 38.33 | 1.08 | **0.03** |
| 2022 | 1413 | 1.73 | 0.67 | 0.04 | **<.01** | 43.67 | 1.11 | **<.01** |
| 2023 | 1633 | 1.72 | 0.65 | -0.01 | 0.95 | 42.44 | 0.99 | 0.71 |
| 2024 | 1522 | 1.75 | 0.64 | 0.01 | 0.36 | 44.88 | 1.04 | 0.23 |
|  |  |  |  |  |  |  |  |  |
| Age (2023- 2020) |  |  |  | -0.01 | <.01 |  | 0.97 | <.01 |
|  |  |  |  |  |  |  |  |  |
| 2024 vs 2020 |  |  |  | 0.07 | **<.01** |  | 1.23 | **<.01** |
|  |  |  |  |  |  |  |  |  |
| **Women, 50-59 years** | | | | | | | | |
| 2020 | 1727 | 1.54 | 0.60 |  |  | 31.21 |  |  |
| 2021 | 1824 | 1.55 | 0.59 | 0.01 | 0.62 | 33.44 | 1.05 | 0.19 |
| 2022 | 1523 | 1.64 | 0.63 | 0.04 | **<.01** | 38.61 | 1.12 | **<.01** |
| 2023 | 1749 | 1.64 | 0.64 | 0.01 | 0.58 | 38.02 | 0.99 | 0.95 |
| 2024 | 1764 | 1.65 | 0.64 | 0.01 | 0.59 | 39.06 | 1.02 | 0.50 |
|  |  |  |  |  |  |  |  |  |
| Age (2023- 2020) |  |  |  | -0.01 | <.01 |  | 0.98 | 0.03 |
|  |  |  |  |  |  |  |  |  |
| 2024 vs 2020 |  |  |  | 0.05 | **<.01** |  | 1.18 | **<.01** |
|  |  |  |  |  |  |  |  |  |
| **Women, 60-69 years** | | | | | | | | |
| 2020 | 1381 | 1.48 | 0.54 |  |  | 27.44 |  |  |
| 2021 | 1459 | 1.49 | 0.53 | 0.003 | 0.77 | 29.27 | 1.04 | 0.32 |
| 2022 | 1316 | 1.55 | 0.56 | 0.03 | **<.01** | 34.19 | 1.12 | **<.01** |
| 2023 | 1478 | 1.51 | 0.54 | -0.02 | 0.11 | 29.43 | .90 | **0.01** |
| 2024 | 1420 | 1.51 | 0.56 | 0.01 | 0.88 | 29.79 | 1.01 | 0.85 |
|  |  |  |  |  |  |  |  |  |
| Age (2023- 2020) |  |  |  | -0.01 | <.01 |  | 0.97 | <.01 |
|  |  |  |  |  |  |  |  |  |
| 2024 vs 2020 |  |  |  | 0.02 | 0.09 |  | 1.05 | .16 |
|  |  |  |  |  |  |  |  |  |
| **Women, 70-79 years** | | | | | | | | |
| 2020 | 823 | 1.42 | 0.47 |  |  | 26.00 |  |  |
| 2021 | 851 | 1.49 | 0.52 | 0.03 | **0.01** | 31.26 | 1.14 | **0.02** |
| 2022 | 777 | 1.50 | 0.51 | 0.01 | 0.62 | 30.12 | 0.97 | 0.59 |
| 2023 | 902 | 1.47 | 0.53 | -0.02 | 0.23 | 26.50 | 0.91 | 0.09 |
| 2024 | 820 | 1.45 | 0.49 | -0.01 | 0.46 | 25.98 | 0.99 | 0.87 |
|  |  |  |  |  |  |  |  |  |
| Age (2023- 2020) |  |  |  | -0.01 | 0.04 |  | 0.98 | 0.13 |
|  |  |  |  |  |  |  |  |  |
| 2024 vs 2020 |  |  |  | 0.01 | 0.28 |  | 1.01 | 0.97 |
|  |  |  |  |  |  |  |  |  |
| **Women, 80-89 years** | | | | | | | | |
| 2020 | 174 | 1.46 | 0.56 |  |  | 24.14 |  |  |
| 2021 | 164 | 1.49 | 0.54 | 0.02 | 0.45 | 29.27 | 1.15 | 0.25 |
| 2022 | 168 | 1.52 | 0.53 | 0.01 | 0.63 | 27.38 | 0.95 | 0.71 |
| 2023 | 200 | 1.56 | 0.57 | 9.92 | 0.50 | 30.00 | 1.07 | 0.55 |
| 2024 | 191 | 1.50 | 0.56 | -0.03 | 0.35 | 25.65 | 0.89 | 0.32 |
|  |  |  |  |  |  |  |  |  |
| Age (2023- 2020) |  |  |  | 0.02 | <.01 |  | 1.06 | 0.05 |
|  |  |  |  |  |  |  |  |  |
| 2024 vs 2020 |  |  |  | 0.02 | 0.38 |  | 1.04 | 0.72 |

**Table 4b.** Mean score and percentage scoring above cut-off level for mental health problems by age group for men in the Quality of life survey

| **Quality of life survey** | | | | | | | | |
| --- | --- | --- | --- | --- | --- | --- | --- | --- |
| **Year** | **N=** | **m=** | **sd=** | **Reg. Coefficient comparing this year to the year prior** | **P=** | **Percentage scoring ≥2.00** | **OR for being above cut of in this year compared to the year prior** | **P=** |
| **Men, 20-29 years** | | | | | | | | |
| 2020 | 1179 | 1.68 | 0.68 |  |  | 31.13 |  |  |
| 2021 | 1087 | 1.78 | 0.70 | 0.05 | **<.01** | 36.06 | 1.12 | **0.01** |
| 2022 | 924 | 1.88 | 0.74 | 0.05 | **<.01** | 40.69 | 1.09 | **0.04** |
| 2023 | 1238 | 1.86 | 0.72 | -0.01 | 0.82 | 40.06 | 1.01 | 0.85 |
| 2024 | 1076 | 1.82 | 0.69 | -0.02 | 0.13 | 35.97 | 0.91 | **0.03** |
|  |  |  |  |  |  |  |  |  |
| Age (2024- 2020) |  |  |  | -0.01 | 0.79 |  | 0.99 | 0.47 |
|  |  |  |  |  |  |  |  |  |
| 2024 vs 2020 |  |  |  | 0.07 | **<.01** |  | 1.12 | **0.01** |
| **Men, 30-39 years** | | | | | | | | |
| 2020 | 1241 | 1.68 | 0.67 |  |  | 29.57 |  |  |
| 2021 | 1204 | 1.70 | 0.70 | 0.01 | 0.32 | 31.89 | 1.06 | 0.20 |
| 2022 | 1049 | 1.74 | 0.70 | 0.02 | 0.20 | 34.32 | 1.05 | 0.25 |
| 2023 | 1407 | 1.75 | 0.70 | 0.01 | 0.50 | 33.40 | 0.98 | 0.80 |
| 2024 | 1283 | 1.77 | 0.69 | 0.01 | 0.56 | 35.78 | 1.05 | 0.22 |
|  |  |  |  |  |  |  |  |  |
| Age (2024- 2020) |  |  |  | -0.01 | 0.03 |  | 0.99 | 0.35 |
|  |  |  |  |  |  |  |  |  |
| 2024 vs 2020 |  |  |  | 0.05 | **<.01** |  | 1.15 | **0.01** |
|  |  |  |  |  |  |  |  |  |
| **Men, 40-49 years** | | | | | | | | |
| **Year** | **N=** | **m=** | **sd=** | **Reg. Coefficient comparing this year to the year prior** | **P=** | **Percentage scoring ≥2.00** | **OR for being above cut of in this year compared to the year prior** | **P=** |
| 2020 | 1546 | 1.54 | 0.61 |  |  | 22.57 |  |  |
| 2021 | 1509 | 1.57 | 0.64 | 0.01 | 0.24 | 24.19 | f | 0.27 |
| 2022 | 1208 | 1.63 | 0.65 | 0.03 | **0.01** | 28.15 | 1.11 | **0.02** |
| 2023 | 1488 | 1.67 | 0.70 | 0.02 | 0.06 | 28.23 | 1.01 | 0.73 |
| 2024 | 1337 | 1.66 | 0.64 | -0.01 | 0.38 | 28.20 | 0.99 | 0.84 |
|  |  |  |  |  |  |  |  |  |
| Age (2024- 2020) |  |  |  | -0.01 | <.01 |  | 0.96 | <.01 |
|  |  |  |  |  |  |  |  |  |
| 2024 vs 2020 |  |  |  | 0.06 | **<.01** |  | 1.16 | **<.01** |
|  |  |  |  |  |  |  |  |  |
| **Men, 50-59 years** | | | | | | | | |
| 2020 | 1739 | 1.46 | 0.57 |  |  | 17.65 |  |  |
| 2021 | 1696 | 1.48 | 0.58 | 0.01 | 0.22 | 20.46 | 1.09 | **0.03** |
| 2022 | 1482 | 1.52 | 0.57 | 0.02 | **0.056** | 21.05 | 1.01 | 0.71 |
| 2023 | 1689 | 1.52 | 0.59 | 0.002 | 0.82 | 21.37 | 1.01 | 0.75 |
| 2024 | 1672 | 1.55 | 0.62 | 0.02 | 0.14 | 23.92 | 1.08 | 0.07 |
|  |  |  |  |  |  |  |  |  |
| Age (2024- 2020) |  |  |  | -0.01 | <.01 |  | 0.96 | <.01 |
|  |  |  |  |  |  |  |  |  |
| 2024 vs 2020 |  |  |  | 0.05 | **<.01** |  | 1.21 | **<.01** |
|  |  |  |  |  |  |  |  |  |
| **Men, 60-69 years** | | | | | | | | |
| 2020 | 1451 | 1.37 | 0.51 |  |  | 13.92 |  |  |
| 2021 | 1521 | 1.38 | 0.52 | 0.01 | 0.55 | 14.00 | 1.01 | 0.97 |
| 2022 | 1317 | 1.41 | 0.52 | 0.02 | 0.11 | 15.41 | 1.05 | 0.28 |
| 2023 | 1449 | 1.42 | 0.54 | 0.01 | 0.61 | 17.60 | 1.09 | 0.11 |
| 2024 | 1517 | 1.44 | 0.53 | 0.01 | 0.28 | 16.94 | 0.98 | 0.75 |
|  |  |  |  |  |  |  |  |  |
| Age (2024- 2020) |  |  |  | -0.01 | <.01 |  | 0.94 | **<.01** |
|  |  |  |  |  |  |  |  |  |
| 2024 vs 2020 |  |  |  | 0.04 | **<.01** |  | 1.12 | **0.02** |
|  |  |  |  |  |  |  |  |  |
| **Men, 70-79 years** | | | | | | | | |
| 2020 | 952 | 1.30 | 0.43 |  |  | 10.40 |  |  |
| 2021 | 1005 | 1.34 | 0.46 | 0.02 | 0.10 | 13.23 | 1.15 | **0.05** |
| 2022 | 920 | 1.34 | 0.42 | 0.01 | 0.77 | 10.87 | 0.89 | 0.11 |
| 2023 | 978 | 1.34 | 0.47 | 0.001 | 0.95 | 12.27 | 1.07 | 0.34 |
| 2024 | 986 | 1.36 | 0.48 | 0.01 | 0.35 | 12.78 | 1.02 | 0.72 |
|  |  |  |  |  |  |  |  |  |
| Age (2024- 2020) |  |  |  | -0.01 | 0.34 |  | 0.99 | 0.68 |
|  |  |  |  |  |  |  |  |  |
| 2024 vs 2020 |  |  |  | 0.03 | **<.01** |  | 1.12 | 0.10 |
|  |  |  |  |  |  |  |  |  |
| **Men, 80-89 years** | | | | | | | | |
| 2020 | 171 | 1.39 | 0.45 |  |  | 14.62 |  |  |
| 2021 | 213 | 1.33 | 0.37 | -0.03 | 0.17 | 10.80 | 0.84 | 0.26 |
| 2022 | 218 | 1.36 | 0.42 | 0.01 | 0.48 | 12.39 | 1.07 | 0.64 |
| 2023 | 212 | 1.36 | 0.45 | 0.01 | 0.91 | 15.09 | 1.12 | 0.41 |
| 2024 | 264 | 1.37 | 0.47 | 0.01 | 0.84 | 12.12 | 0.88 | 0.36 |
|  |  |  |  |  |  |  |  |  |
| Age (2024- 2020) |  |  |  | 0.01 | 0.66 |  | 1.01 | 0.75 |
|  |  |  |  |  |  |  |  |  |
| 2024 vs 2020 |  |  |  | -0.01 | 0.80 |  | 0.90 | 0.47 |

**Table 5a.** Mean score and percentage scoring above cut-off level for mental health problems by age group for female students in the Student’s Health and Wellbeing Study (SHoT)

| **SHoT**  **Female student 18-28 years old** | | | | | | | | |
| --- | --- | --- | --- | --- | --- | --- | --- | --- |
| **Year** | **N=** | **m=** | **sd=** | **Reg. Coefficient comparing this year to the year prior** | **P=** | **Percentage scoring ≥2.75*** | **OR for being above cut of in this year compared to the year prior** | **P=** |
| 2010 | 3934 | 1.85 | 0.71 |  |  | 13.42 |  |  |
| 2014 | 8221 | 1.98 | 0.72 | 0.07 | **<.01** | 16.59 | 1.13 | **<.01** |
| 2018 | 33477 | 2.12 | 0.79 | 0.07 | **<.01** | 24.00 | 1.26 | **<.01** |
| 2021 | 36610 | 2.42 | 0.77 | 0.15 | **<.01** | 35.39 | 1.32 | **<.01** |
| 2022 | 32306 | 2.18 | 0.81 | -0.12 | **<.01** | 27.05 | 0.82 | **<.01** |
|  |  |  |  |  |  |  |  |  |
| 2022 vs 2010 |  |  |  | 0.17 | **<.01** |  | 1.55 | **<.01** |

*Recommended cutt-off levels for female students in the Norwegian student population

* Regression analyses for SHoT are weighted to take the differing n between data-collections into account.

**Table 5b.** Mean score and percentage scoring above cut-off level for mental health problems by age group for male students in the Student’s Health and Wellbeing Study (SHoT)

| **SHoT**  **Male student 18-28 years old** | | | | | | | | |
| --- | --- | --- | --- | --- | --- | --- | --- | --- |
| **Year** | **N=** | **m=** | **sd=** | **Reg. Coefficient comparing this year to the year prior** | **P=** | **Percentage scoring ≥2.25*** | **OR for being above cut of in this year compared to the year prior** | **P=** |
| 2010 | 2037 | 1.57 | 0.63 |  |  | 12.67 |  |  |
| 2014 | 3999 | 1.66 | 0.64 | 0.04 | **<.01** | 15.73 | 1.13 | **<.01** |
| 2018 | 14815 | 1.74 | 0.72 | 0.04 | **<.01** | 20.57 | 1.17 | **<.01** |
| 2021 | 19098 | 2.02 | 0.75 | 0.14 | **<.01** | 32.06 | 1.35 | **<.01** |
| 2022 | 15966 | 1.81 | 0.75 | -0.11 | **<.01** | 23.80 | 0.81 | **<.01** |
|  |  |  |  |  |  |  |  |  |
| 2022 vs 2010 |  |  |  | -0.12 | **<.01** |  | 1.47 | **<.01** |

*Recommended cutt-off levels for female students in the Norwegian student population

* Regression analyses for SHoT are weighted to take the differing n between data-collections into account.

**Table 6a.** Mean score and percentage scoring above cut-off level for mental health problems by age group for women in the Tromsø study

| **Tromsø Survey** | | | | | | | | |
| --- | --- | --- | --- | --- | --- | --- | --- | --- |
| **Year** | **N=** | **m=** | **sd=** | **Reg. Coefficient comparing this year to the year prior** | **P=** | **Percentage scoring ≥1.85** | **OR for being above cut of in this year compared to the year prior** | **P=** |
| **Women, 30-39 years** | | | | | | | | |
| 2001 | 404 | 1.29 | 0.39 |  |  | 8.91 |  |  |
| 2007-08 | 291 | 1.39 | 0.50 | 0.06 | **<.01** | 13.40 | 1.33 | **0.05** |
|  |  |  |  |  |  |  |  |  |
| Age (2001-2007-08) |  |  |  | -0.01 | 0.13 |  | 0.97 | 0.46 |
|  |  |  |  |  |  |  |  |  |
| **Women, 40-49 years** | | | | | | | | |
| 2001 | 733 | 1.32 | 0.43 |  |  | 9.96 |  |  |
| 2007-08 | 1877 | 1.34 | 0.40 | -0.01 | 0.65 | 10.66 | 0.95 | 0.42 |
| 2015-16 | 3304 | 1.39 | 0.45 | 0.03 | **<.01** | 13.80 | 1.16 | **<.01** |
|  |  |  |  |  |  |  |  |  |
| Age (2001- 2015-16) |  |  |  | 0.01 | 0.16 |  | 1.02 | 0.19 |
|  |  |  |  |  |  |  |  |  |
| 2001 vs. 2015-16 |  |  |  | 0.04 | **<.01** |  | 1.22 | **<.01** |
|  |  |  |  |  |  |  |  |  |
| **Women, 50-59 years** | | | | | | | | |
| 2001 | 650 | 1.32 | 0.39 |  |  | 10.62 |  |  |
| 2007-08 | 1249 | 1.35 | 0.42 | 0.01 | 0.19 | 11.29 | 1.02 | 0.83 |
| 2015-16 | 3163 | 1.36 | 0.42 | 0.01 | 0.33 | 11.95 | 1.04 | 0.45 |
|  |  |  |  |  |  |  |  |  |
|  |  |  |  |  |  |  |  |  |
| Age (2001- 2015-16) |  |  |  | 0.00 | 0.99 |  | 1.01 | 0.67 |
|  |  |  |  |  |  |  |  |  |
| 2001 vs. 2015-16 |  |  |  | 0.02 | **0.02** |  | 1.08 | 0.26 |
|  |  |  |  |  |  |  |  |  |
| **Women, 60-69 years** | | | | | | | | |
| 2001 | 1193 | 1.29 | 0.41 |  |  | 8.80 |  |  |
| 2007-08 | 1946 | 1.33 | 0.41 | 0.02 | **0.02** | 10.53 | 1.09 | 0.15 |
| 2015-16 | 2594 | 1.32 | 0.38 | -0.002 | 0.64 | 9.79 | 0.97 | 0.61 |
|  |  |  |  |  |  |  |  |  |
| Age (2001- 2015-16) |  |  |  | -0.01 | <.01 |  | 0.95 | <.01 |
|  |  |  |  |  |  |  |  |  |
| 2001 vs. 2015-16 |  |  |  | 0.01 | 0.11 |  | 1.05 | 0.43 |
|  |  |  |  |  |  |  |  |  |
| **Women, 70-79 years** | | | | | | | | |
| 2001 | 770 | 1.29 | 0.37 |  |  | 7.92 |  |  |
| 2007-08 | 827 | 1.34 | 0.39 | 0.03 | **<.01** | 10.28 | 1.20 | 0.056 |
| 2015-16 | 1264 | 1.26 | 0.31 | -0.04 | **<.01** | 4.98 | 0.68 | **<.01** |
|  |  |  |  |  |  |  |  |  |
|  |  |  |  |  |  |  |  |  |
| Age (2001- 2015-16) |  |  |  | 0.002 | 0.37 |  | 1.01 | 0.64 |
|  |  |  |  |  |  |  |  |  |
| 2001 vs. 2015-16 |  |  |  | -0.02 | **0.03** |  | 0.78 | **<.01** |
|  |  |  |  |  |  |  |  |  |
| **Women, 80-89 years** |  |  |  |  |  |  |  |  |
| 2001 | 93 |  |  |  |  |  |  |  |
| 2007-08 | 258 | 1.43 | 0.44 | 0.06 |  | 15.89 | 1.41 |  |
| 2015-16 | 338 | 1.29 | 0.35 | -0.08 | **<.01** | 8.28 | 0.67 | **<.01** |
| Age (2001- 2015-16) |  |  |  | 0.02 | 0.02 |  | 1.10 | 0.05 |

*The Tromsø survey also included participants aged 80-89 years old in 2001. However, we only include data for participants aged 80-89 for surveys that had close to n>100 for each time point. The Tromsø survey in 2001 did not fulfill this criteria, and consequently only data from 2007 and 2015 are included for participants 8-89 years.

**Table 6b.** Mean score and percentage scoring above cut-off level for mental health problems by age group for men in the Tromsø study

| **Tromsø study** | | | | | | | | |
| --- | --- | --- | --- | --- | --- | --- | --- | --- |
| **Year** | **N=** | **m=** | **sd=** | **Reg. Coefficient comparing this year to the year prior** | **P=** | **Percentage scoring ≥1.85** | **OR for being above cut of in this year compared to the year prior** | **P=** |
| **Men, 30-39 years** | | | | | | | | |
| 2001 | 272 | 1.19 | 0.29 |  |  | 2.21 |  |  |
| 2007-08 | 208 | 1.26 | 0.31 | 0.03 | 0.07 | 6.73 | 1.53 | 0.14 |
|  |  |  |  |  |  |  |  |  |
| Age (2001-2007-08) |  |  |  | 0.01 | 0.58 |  | 1.09 | 0.29 |
|  |  |  |  |  |  |  |  |  |
| **Men, 40-49 years** | | | | | | | | |
| 2001 | 585 | 1.21 | 0.31 |  |  | 5.30 |  |  |
| 2007-08 | 1644 | 1.25 | 0.36 | 0.01 | 0.49 | 7.24 | 1.03 | 0.71 |
| 2015-16 | 2989 | 1.29 | 0.39 | 0.02 | **<.01** | 8.77 | 1.13 | 0.41 |
|  |  |  |  |  |  |  |  |  |
| Age (2001- 2015-16) |  |  |  | 0.00 | 0.98 |  | 0.99 | 0.69 |
|  |  |  |  |  |  |  |  |  |
| 2001 vs. 2015-16 |  |  |  | 0.04 | **<.01** |  | 1.29 | **<.01** |
|  |  |  |  |  |  |  |  |  |
| **Men, 50-59 years** | | | | | | | | |
| 2001 | 340 | 1.19 | 0.29 |  |  | 4.41 |  |  |
| 2007-08 | 1121 | 1.27 | 0.38 | 0.03 | **0.01** | 7.58 | 1.09 | 0.38 |
| 2015-16 | 2730 | 1.26 | 0.38 | 0.001 | 0.81 | 8.39 | 1.07 | 0.29 |
|  |  |  |  |  |  |  |  |  |
| Age (2001- 2015-16) |  |  |  | -0.003 | 0.13 |  | 0.96 | 0.09 |
|  |  |  |  |  |  |  |  |  |
| 2001 vs. 2015-16 |  |  |  | 0.03 | **0.01** |  | 1.24 | **0.03** |
|  |  |  |  |  |  |  |  |  |
| **Men, 60-69 years** | | | | | | | | |
| 2001 | 1137 | 1.17 | 0.28 |  |  | 3.25 |  |  |
| 2007-08 | 1926 | 1.19 | 0.28 | 0.01 | 0.22 | 4.52 | 1.15 | 0.14 |
| 2015-16 | 2444 | 1.19 | 0.29 | 0.004 | 0.28 | 4.21 | 0.98 | 0.84 |
|  |  |  |  |  |  |  |  |  |
| Age (2001- 2015-16) |  |  |  | -0.01 | <.01 |  | 0.93 | <.01 |
|  |  |  |  |  |  |  |  |  |
| 2001 vs. 2015-16 |  |  |  | 0.01 | **0.02** |  | 1.11 | 0.24 |
|  |  |  |  |  |  |  |  |  |
| **Men, 70-79 years** | | | | | | | | |
| **Year** | **N=** | **m=** | **sd=** | **Reg. Coefficient comparing this year to the year prior** | **P=** | **Percentage scoring ≥1.85** | **OR for being above cut of in this year compared to the year prior** | **P=** |
| 2001 | 749 | 1.18 | 0.28 |  |  | 3.20 |  |  |
| 2007-08 | 779 | 1.21 | 0.31 | 0.02 | **0.03** | 4.75 | 1.26 | 0.09 |
| 2015-16 | 1272 | 1.17 | 0.25 | -0.02 | **<.01** | 2.75 | 0.75 | **0.02** |
|  |  |  |  |  |  |  |  |  |
| Age (2001- 2015-16) |  |  |  | 0.003 | 0.13 |  | 0.99 | 0.87 |
|  |  |  |  |  |  |  |  |  |
| 2001 vs. 2015-16 |  |  |  | -0.01 | 0.14 |  | 0.89 | 0.38 |
|  |  |  |  |  |  |  |  |  |
| **Men, 80-89 years** |  |  |  |  |  |  |  |  |
| 2001 | 95 |  |  |  |  |  |  |  |
| 2007-08 | 167 | 1.22 | 0.31 | 0.01 |  | 5.39 | 1.16 |  |
| 2015-16 | 291 | 1.19 | 0.32 | -0.01 | 0.45 | 4.47 | 0.91 | 0.68 |
|  |  |  |  |  |  |  |  |  |
| Age (2001- 2015-16) |  |  |  | 4.90 | 0.99 |  | 0.99 | 0.98 |

*The Tromsø survey also included participants aged 80-89 years old in 2001. However, we only include data for participants aged 80-89 for surveys that had close to n>100 for each time point. The Tromsø survey in 2001 did not fulfill this criteria, and consequently only data from 2007 and 2015 are included for participants 8-89 years.

**Table 7a.** Mean score and percentage scoring above cut-off level for mental health problems by age group for women in the SAMINOR study

| **SAMINOR** | | | | | | | | |
| --- | --- | --- | --- | --- | --- | --- | --- | --- |
| **Year** | **N=** | **m=** | **sd=** | **Reg. Coefficient comparing this year to the year prior** | **P=** | **Percentage scoring ≥ 1.85** | **OR for being above cut of in this year compared to the year prior** | **P=** |
| **Women 40-49 years old** | | | | | | | | |
| 2003-04 | 2152 | 1.35 | 0.44 |  |  | 11.66 |  |  |
| 2012 | 1258 | 1.38 | 0.48 | 0.03 | **0.03** | 13.83 | 1.22 | 0.07 |
| Age (2003-2012) |  |  |  | 0.01 | 0.77 |  | 1.00 | 0.93 |
|  |  |  |  |  |  |  |  |  |
| **Women 50-59 years old** | | | | | | | | |
| 2003-04 | 2218 | 1.36 | 0.46 |  |  | 12.58 |  |  |
| 2012 | 1267 | 1.34 | 0.45 | -0.02 | 0.27 | 11.21 | 0.88 | 0.24 |
| Age (2003-2012) |  |  |  | -0.01 | 0.24 |  | 0.98 | 0.32 |
|  |  |  |  |  |  |  |  |  |
| **Women 60-69 years old** | | | | | | | | |
| 2003-04 | 1357 | 1.28 | 0.37 |  |  | 7.07 |  |  |
| 2012 | 1035 | 1.28 | 0.38 | 0.01 | 0.88 | 8.50 | 1.21 | 0.22 |
| Age (2003-2021) |  |  |  | -0.01 | <.01 |  | 0.90 | <0.01 |
|  |  |  |  |  |  |  |  |  |

**Table 7a.** Mean score and percentage scoring above cut-off level for mental health problems by age group for men in the SAMINOR study

| **SAMINOR** | | | | | | | | |
| --- | --- | --- | --- | --- | --- | --- | --- | --- |
| **Year** | **N=** | **m=** | **sd=** | **Reg. Coefficient comparing this year to the year prior** | **P=** | **Percentage scoring ≥1.85** | **OR for being above cut of in this year compared to the year prior** | **P=** |
| **Men 40-49 years old** | | | | | | | | |
| 2003-04 | 1897 | 1.27 | 0.42 |  |  | 8.17 |  |  |
| 2012 | 917 | 1.27 | 0.46 | 0.01 | 0.76 | 9.16 | 1.13 | 0.38 |
| Age (2003-2012) |  |  |  | 0.01 | 0.33 |  | 1.01 | 0.56 |
|  |  |  |  |  |  |  |  |  |
| **Men 50-59 years old** | | | | | | | | |
| 2003-04 | 2229 | 1.26 | 0.39 |  |  | 7.85 |  |  |
| 2012 | 1118 | 1.27 | 0.41 | 0.01 | 0.49 | 8.14 | 1.04 | 0.78 |
| Age (2003-2012) |  |  |  | 0.01 | 0.44 |  | 1.00 | 0.88 |
|  |  |  |  |  |  |  |  |  |
| **Men 60-69 years old** | | | | | | | | |
| 2003-04 | 1451 | 1.19 | 0.31 |  |  | 4.55 |  |  |
| 2012 | 1181 | 1.20 | 0.32 | 0.01 | 0.48 | 6.10 | 1.37 | 0.07 |
| Age (2003-2012) |  |  |  | -0.01 | 0.01 |  | 0.97 | 0.28 |
|  |  |  |  |  |  |  |  |  |

**Table 8a.** Mean score and percentage scoring above cut-off level for mental health problems by age group for women in FHUS Oslo

| **FHUS Oslo** | | | | | | | | |
| --- | --- | --- | --- | --- | --- | --- | --- | --- |
| **Year** | **N=** | **m=** | **sd=** | **Reg. Coefficient comparing this year to the year prior** | **P=** | **Percentage scoring ≥1.80** | **OR for being above cut of in this year compared to the year prior** | **P=** |
| **Women, 20-29 years** | | | | | | | | |
| 2020 | 953 | 2.08 | 0.81 |  |  | 60.76 |  |  |
| 2021 | 530 | 2.08 | 0.80 | 0.01 | 0.89 | 60.94 | 1.01 | 0.80 |
| 2024 | 5 037 | 2.08 | 0.78 | 0.01 | 0.95 | 62.95 | 1.04 | 0.45 |
| Age (2020-2024) |  |  |  | -0.04 | <.01 |  | 0.92 | <.01 |
|  |  |  |  |  |  |  |  |  |
| 2020 vs. 2024 |  |  |  | 0.01 | 0.77 |  | 1.05 | 0.16 |
|  |  |  |  |  |  |  |  |  |
| **Women, 30-39 years** | | | | | | | | |
| 2020 | 1099 | 1.87 | 0.73 |  |  | 51.87 |  |  |
| 2021 | 925 | 1.81 | 0.72 | -0.03 | 0.11 | 45.30 | 0.89 | **<0.01** |
| 2024 | 5 179 | 1.93 | 0.74 | 0.06 | **<.01** | 53.89 | 1.19 | **<.01** |
| Age (2020-2024) |  |  |  | -0.01 | 0.01 |  | 0.97 | 0.02 |
|  |  |  |  |  |  |  |  |  |
| 2020 vs. 2024 |  |  |  | 0.03 | **0.01** |  | 1.04 | 0.18 |
|  |  |  |  |  |  |  |  |  |
| **Women, 40-49 years** | | | | | | | | |
| 2020 | 920 | 1.71 | 0.67 |  |  | 42.72 |  |  |
| 2021 | 815 | 1.67 | 0.68 | -0.01 | 0.38 | 38.04 | 0.92 | 0.07 |
| 2024 | 4 211 | 1.81 | 0.71 | 0.07 | **<.01** | 46.62 | 1.19 | **<.01** |
| Age (2020-2024) |  |  |  | -0.01 | <.01 |  | 0.96 | <.01 |
|  |  |  |  |  |  |  |  |  |
| 2020 vs. 2024 |  |  |  | 0.05 | **<.01** |  | 1.08 | **0.05** |
|  |  |  |  |  |  |  |  |  |
| **Year** | **N=** | **m=** | **sd=** | **Reg. Coefficient comparing this year to the year prior** | **P=** | **Percentage scoring ≥1.80** | **OR for being above cut of in this year compared to the year prior** | **P=** |
| **Women, 50-59 years** | | | | | | | | |
| 2020 | 872 | 1.66 | 0.60 |  |  | 39.56 |  |  |
| 2021 | 812 | 1.58 | 0.60 | -0.04 | **0.02** | 32.51 | 0.87 | <.01 |
| 2024 | 4 061 | 1.69 | 0.65 | 0.06 | **<.01** | 40.63 | 1.19 | <.01 |
| Age (2020-2024) |  |  |  | -0.01 | <.01 |  | 0.97 | 0.03 |
|  |  |  |  |  |  |  |  |  |
| 2020 vs. 2024 |  |  |  | 0.02 | 0.09 |  | 1.02 | 0.54 |
|  |  |  |  |  |  |  |  |  |
| **Women, 60-69 years** | | | | | | | | |
| 2020 | 728 | 1.56 | 0.56 |  |  | 32.55 |  |  |
| 2021 | 668 | 1.47 | 0.52 | -0.4 | **<.01** | 27.50 | 0.88 | **0.04** |
| 2024 | 3 133 | 1.52 | 0.58 | 0.03 | **0.02** | 29.17 | 1.04 | 0.36 |
| Age (2020-2024) |  |  |  | -0.01 | 0.19 |  | 0.97 | 0.12 |
|  |  |  |  |  |  |  |  |  |
| 2020 vs. 2024 |  |  |  | -0.02 | 0.18 |  | 0.93 | 0.09 |
|  |  |  |  |  |  |  |  |  |
| **Women, 70-79 years** | | | | | | | | |
| 2020 | 322 | 1.57 | 0.55 |  |  | 35.71 |  |  |
| 2021 | 306 | 1.54 | 0.56 | -0.02 | 0.41 | 31.70 | 0.89 | 0.22 |
| 2024 | 1 968 | 1.47 | 0.53 | -0.03 | **0.03** | 24.95 | 0.84 | **0.01** |
| Age (2020-2024) |  |  |  | -0.01 | 0.96 |  | 0.98 | 0.57 |
|  |  |  |  |  |  |  |  |  |
| 2020 vs. 2024 |  |  |  | -0.05 | **<.01** |  | 0.78 | **<.01** |

* Regression analyses for FHUS Oslo are weighted to take the differing n between data-collections into account.

*The FHUS Oslo also included participants aged 80-89 years old. However, we only include data for participants aged 80-89 for surveys that had close to n>100 for at least two time points. The FHUS Oslo in 2020 and 2021 did not fulfill this criteria, and consequently only data for participants 8-89 years are not included for FHUS Oslo.

**Table 8b.** Mean score and percentage scoring above cut-off level for mental health problems by age group for men in FHUS Oslo

| **FHUS Oslo** | | | | | | | | |
| --- | --- | --- | --- | --- | --- | --- | --- | --- |
| **Year** | **N=** | **m=** | **sd=** | **Reg. Coefficient comparing this year to the year prior** | **P=** | **Percentage scoring ≥2.00** | **OR for being above cut of in this year compared to the year prior** | **P=** |
| **Men, 20-29 years** | | | | | | | | |
| 2020 | 524 | 1.77 | 0.69 |  |  | 34.54 |  |  |
| 2021 | 309 | 1.83 | 0.76 | 0.06 | **0.03** | 36.89 | 1.13 | 0.09 |
| 2024 | 3 007 | 1.92 | 0.75 | 0.04 | 0.06 | 43.40 | 1.14 | **0.04** |
| Age (2020-2024) |  |  |  | -0.02 | **0.01** |  | 0.95 | **0.01** |
|  |  |  |  |  |  |  |  |  |
| 2020 vs. 2024 |  |  |  | 0.08 | **<.01** |  | 1.20 | **<.01** |
|  |  |  |  |  |  |  |  |  |
| **Men, 30-39 years** | | | | | | | | |
| 2020 | 846 | 1.67 | 0.69 |  |  | 29.43 |  |  |
| 2021 | 723 | 1.65 | 0.69 | 0.01 | 0.74 | 27.80 | 0.99 | 0.94 |
| 2024 | 3 978 | 1.85 | 0.74 | 0.10 | <.01 | 39.92 | 1.31 | <0.01 |
| Age (2020-2024) |  |  |  | -0.01 | 0.21 |  | 0.97 | 0.02 |
|  |  |  |  |  |  |  |  |  |
| 2020 vs. 2024 |  |  |  | 0.09 | **<.01** |  | 1.27 | **<.01** |
|  |  |  |  |  |  |  |  |  |
| **Men, 40-49 years** | | | | | | | | |
| 2020 | 789 | 1.57 | 0.65 |  |  | 24.71 |  |  |
| 2021 | 689 | 1.55 | 0.60 | -0.01 | 0.79 | 21.48 | 0.95 | 0.38 |
| 2024 | 3 423 | 1.77 | 0.73 | 0.11 | **<.01** | 35.26 | 1.41 | **<.01** |
| Age (2020-2024) |  |  |  | -0.02 | <.01 |  | 0.96 | <.01 |
|  |  |  |  |  |  |  |  |  |
| 2020 vs. 2024 |  |  |  | 0.10 | **<.01** |  | 1.28 | **<.01** |
|  |  |  |  |  |  |  |  |  |
| **Year** | **N=** | **m=** | **sd=** | **Reg. Coefficient comparing this year to the year prior** | **P=** | **Percentage scoring ≥2.00** | **OR for being above cut of in this year compared to the year prior** | **P=** |
| **Men, 50-59 years** | | | | | | | | |
| 2020 | 753 | 1.50 | 0.61 |  |  | 21.12 |  |  |
| 2021 | 713 | 1.45 | 0.57 | -0.02 | 0.21 | 18.65 | 0.95 | 0.36 |
| 2024 | 3 533 | 1.62 | 0.66 | 0.09 | **<.01** | 28.96 | 1.33 | **<0.01** |
| Age (2020-2024) |  |  |  | -0.01 | 0.04 |  | 0.97 | 0.09 |
|  |  |  |  |  |  |  |  |  |
| 2020 vs. 2024 |  |  |  | 0.06 | **<.01** |  | 1.23 | **<.01** |
|  |  |  |  |  |  |  |  |  |
| **Men, 60-69 years** | | | | | | | | |
| 2020 | 547 | 1.39 | 0.52 |  |  | 16.82 |  |  |
| 2021 | 567 | 1.34 | 0.49 | -0.02 | 0.14 | 12.17 | 0.85 | **0.04** |
| 2024 | 2 680 | 1.45 | 0.58 | 0.05 | **<.01** | 18.99 | 1.31 | **<.01** |
| Age (2020-2024) |  |  |  | -0.01 | <.01 |  | 0.94 | 0.01 |
|  |  |  |  |  |  |  |  |  |
| 2020 vs. 2024 |  |  |  | 0.03 | **0.01** |  | 1.08 | 0.19 |
|  |  |  |  |  |  |  |  |  |
| **Men, 70-79 years** | | | | | | | | |
| 2020 | 312 | 1.29 | 0.40 |  |  | 9.62 |  |  |
| 2021 | 306 | 1.23 | 0.35 | -0.03 | 0.11 | 6.86 | 0.88 | 0.31 |
| 2024 | 1 879 | 1.34 | 0.47 | 0.05 | **<.01** | 12.56 | 1.41 | **<.01** |
| Age (2020-2024) |  |  |  | -0.01 | 0.78 |  | 1.01 | 0.84 |
|  |  |  |  |  |  |  |  |  |
| 2020 vs. 2024 |  |  |  | 0.02 | 0.09 |  | 1.16 | 0.16 |

*The FHUS Oslo also included participants aged 80-89 years old. However, we only include data for participants aged 80-89 for surveys that had n>100 for at least two time points. The FHUS Oslo in 2020 and 2021 did not fulfill this criteria, and consequently only data for participants 8-89 years are not included for FHUS Oslo.

**Table 9a.** Mean score and percentage scoring above cut-off level for mental health problems by age group for women in FHUS Agder

| **FHUS Agder** | | | | | | | | |
| --- | --- | --- | --- | --- | --- | --- | --- | --- |
| **Year** | **N=** | **m=** | **sd=** | **Reg. Coefficient comparing this year to the year prior** | **P=** | **Percentage scoring ≥1.80** | **OR for being above cut of in this year compared to the year prior** | **P=** |
| **Women, 20-29 years** | | | | | | | | |
| 2019 | 2 532 | 1.84 | 0.74 |  |  | 48.89 |  |  |
| 2023 | 1 174 | 2.03 | 0.81 | 0.10 | **<.01** | 57.92 | 1.21 | **<.01** |
| Age |  |  |  | -0.04 | <.01 |  | 0.93 | <.01 |
|  |  |  |  |  |  |  |  |  |
| **Women, 30-39 years** | | | | | | | | |
| 2019 | 2 578 | 1.63 | 0.66 |  |  | 35.80 |  |  |
| 2023 | 1 647 | 1.83 | 0.75 | 0.10 | **<.01** | 48.27 | 1.29 | **<.01** |
| Age |  |  |  | -0.02 | <.01 |  | 0.95 | <.01 |
|  |  |  |  |  |  |  |  |  |
| **Women, 40-49 years** | | | | | | | | |
| 2019 | 2 994 | 1.53 | 0.58 |  |  | 29.73 |  |  |
| 2023 | 1 847 | 1.68 | 0.69 | 0.08 | **<.01** | 39.52 | 1.24 | **<.01** |
| Age |  |  |  | -0.01 | <.01 |  | 0.96 | <.01 |
|  |  |  |  |  |  |  |  |  |
| **Women, 50-59 years** | | | | | | | | |
| 2019 | 2 929 | 1.48 | 0.57 |  |  | 26.90 |  |  |
| 2023 | 2 212 | 1.54 | 0.63 | 0.03 | **<.01** | 30.06 | 1.08 | **0.01** |
| Age |  |  |  | -0.01 | 0.50 |  | 0.99 | 0.17 |
|  |  |  |  |  |  |  |  |  |
|  |  |  |  |  |  |  |  |  |
|  |  |  |  |  |  |  |  |  |
|  |  |  |  |  |  |  |  |  |
| **Women, 60-69 years** | | | | | | | | |
| 2019 | 2 268 | 1.37 | 0.48 |  |  | 19.93 |  |  |
| 2023 | 1 837 | 1.42 | 0.53 | 0.03 | **<.01** | 23.57 | 1.12 | **<.01** |
| Age |  |  |  | -0.02 | <.01 |  | 0.95 | <.01 |
|  |  |  |  |  |  |  |  |  |
| **Women, 70-79 years** | | | | | | | | |
| 2019 | 911 | 1.38 | 0.46 |  |  | 20.86 |  |  |
| 2023 | 1 110 | 1.36 | 0.46 | -0.01 | 0.20 | 17.75 | 0.90 | 0.08 |
| Age |  |  |  | 0.01 | 0.81 |  | 1.01 | 0.91 |
|  |  |  |  |  |  |  |  |  |
| **Women, 80-89 years** |  |  |  |  |  |  |  |  |
| 2019 | 106 | 1.34 | 0.43 |  |  | 20.75 |  |  |
| 2023 | 167 | 1.41 | 0.48 | 0.04 | 0.22 | 20.96 | 1.02 | 0.90 |
| Age |  |  |  | -0.01 | 0.37 |  | 0.97 | 0.64 |

**Table 9b.** Mean score and percentage scoring above cut-off level for mental health problems by age group for men in FHUS Agder

| **FHUS Agder** | | | | | | | | |
| --- | --- | --- | --- | --- | --- | --- | --- | --- |
| **Year** | **N=** | **m=** | **sd=** | **Reg. Coefficient comparing this year to the year prior** | **P=** | **Percentage scoring ≥2.00** | **OR for being above cut of in this year compared to the year prior** | **P=** |
| **Men, 20-29 years** | | | | | | | | |
| 2019 | 1 728 | 1.67 | 0.68 |  |  | 29.92 |  |  |
| 2023 | 601 | 1.84 | 0.80 | 0.09 | **<.01** | 38.10 | 1.20 | **<.01** |
| Age |  |  |  | -0.01 | 0.05 |  | 0.98 | 0.31 |
|  |  |  |  |  |  |  |  |  |
| **Men, 30-39 years** | | | | | | | | |
| 2019 | 1 920 | 1.61 | 0.69 |  |  | 25.21 |  |  |
| 2023 | 1 036 | 1.76 | 0.78 | 0.07 | **<.01** | 33.49 | 1.23 | **<.01** |
| Age |  |  |  | -0.01 | <.01 |  | 0.96 | <.01 |
|  |  |  |  |  |  |  |  |  |
| **Men, 40-49 years** | | | | | | | | |
| 2019 | 2 547 | 1.50 | 0.61 |  |  | 20.73 |  |  |
| 2023 | 1 360 | 1.62 | 0.69 | 0.06 | **<.01** | 27.21 | 1.20 | **<.01** |
| Age |  |  |  | -0.01 | <.01 |  | 0.98 | 0.13 |
|  |  |  |  |  |  |  |  |  |
| **Men, 50-59 years** | | | | | | | | |
| 2019 | 2 658 | 1.40 | 0.55 |  |  | 16.67 |  |  |
| 2023 | 1 678 | 1.49 | 0.63 | 0.04 | **<.01** | 20.80 | 1.15 | **<.01** |
| Age |  |  |  | -0.01 | 0.01 |  | 0.97 | 0.02 |
|  |  |  |  |  |  |  |  |  |
|  |  |  |  |  |  |  |  |  |
|  |  |  |  |  |  |  |  |  |
| **Year** | **N=** | **m=** | **sd=** | **Reg. Coefficient comparing this year to the year prior** | **P=** | **Percentage scoring ≥2.00** | **OR for being above cut of in this year compared to the year prior** | **P=** |
| **Men, 60-69 years** | | | | | | | | |
| 2019 | 2 467 | 1.32 | 0.48 |  |  | 12.16 |  |  |
| 2023 | 1 770 | 1.36 | 0.52 | 0.02 | **<.01** | 13.28 | 1.06 | 0.23 |
| Age |  |  |  | -0.01 | <.01 |  | 0.94 | <.01 |
|  |  |  |  |  |  |  |  |  |
| **Men, 70-79 years** | | | | | | | | |
| 2019 | 1 210 | 1.25 | 0.38 |  |  | 7.85 |  |  |
| 2023 | 1 341 | 1.27 | 0.40 | 0.01 | 0.09 | 7.98 | 1.02 | 0.81 |
| Age |  |  |  | -0.01 | 0.56 |  | 0.96 | 0.23 |
|  |  |  |  |  |  |  |  |  |
| **Men, 80-89 years** |  |  |  |  |  |  |  |  |
| 2019 | 182 | 1.30 | 0.45 |  |  | 9.89 |  |  |
| 2023 | 226 | 1.30 | 0.43 | -0.01 | 0.92 | 9.73 | 0.99 | 0.99 |
| Age |  |  |  | -0.01 | 0.96 |  | 0.97 | 0.63 |
